# Determining predictors of sepsis at triage among children under 5 years of age in resource-limited settings: A modified Delphi process

**DOI:** 10.1371/journal.pone.0211274

**Published:** 2019-01-28

**Authors:** Jollee S. T. Fung, Samuel Akech, Niranjan Kissoon, Matthew O. Wiens, Mike English, J. Mark Ansermino

**Affiliations:** 1 Centre for International Child Health, BC Children’s Hospital, Vancouver, BC, Canada; 2 Faculty of Medicine, University of British Columbia, Vancouver, BC, Canada; 3 CanadaHealth Services Unit, KEMRI/Wellcome Trust, Nairobi, Kenya; 4 Department of Pediatrics and Emergency Medicine, University of British Columbia, Vancouver, BC; 5 Department of Anesthesiology, Pharmacology and Therapeutics, University of British Columbia, Vancouver, BC; 6 CanadaSchool of Population and Public Health, University of British Columbia, Vancouver, BC, Canada; Radboud University Medical Center, NETHERLANDS

## Abstract

Sepsis is a life-threatening dysfunction of the immune system leading to multiorgan failure that is precipitated by infectious diseases and is a leading cause of death in children under 5 years of age. It is necessary to be able to identify a sick child at risk of developing sepsis at the earliest point of presentation to a healthcare facility so that appropriate care can be provided as soon as possible. Our study objective was to generate a list of consensus-driven predictor variables for the derivation of a prediction model that will be incorporated into a mobile device and operated by low-skilled healthcare workers at triage. By conducting a systematic literature review and examination of global guideline documents, a list of 72 initial candidate predictor variables was generated. A two-round modified Delphi process involving 26 experts from both resource-rich and resource-limited settings, who were also encouraged to suggest new variables, yielded a final list of 45 predictor variables after evaluating each variable based on three domains: predictive potential, measurement reliability, and level of training and resources required. The final list of predictor variables will be used to collect data and contribute to the derivation of a prediction model.

## Introduction

Infectious diseases, primarily pneumonia, malaria, and diarrheal illnesses, constitute the major causes of post-neonatal deaths in children younger than 5 years of age [1]. According to the Global Burden of Disease Study in 2016, among the 4.9 million deaths in children under 5 years of age, infectious diseases accounted at least 50% of these deaths. [2] The final common pathway to death from most infectious etiologies is sepsis which is defined as a dysregulated immune response leading to multiorgan dysfunction [3]. A recent meta-analysis of population-based neonatal and pediatric sepsis epidemiology extrapolated approximately 3.0 million cases of neonatal sepsis and 1.2 million cases of pediatric sepsis annually, highlighting the immense burden of sepsis as an ultimate outcome of infectious diseases. [4] As most of these deaths occur in resource-limited countries, low socio-economic status and geographic barriers to healthcare facilities represent the major factors associated with mortality due to sepsis. [5] At the 2017 World Health Assembly, sepsis was recognized as a global health priority for the World Health Organization (WHO) [6–8]. An important aspect of the resolution is the promotion of research aimed at improving timely prediction, diagnosis, and treatment of sepsis.

Timely prediction can be elusive without appreciation of the myriad of signs and symptoms triggered by infections that could signify the evolution of sepsis in a sick child. This may present as constellations of cardiac failure, respiratory distress, septic shock, or other organ dysfunction, together representing the syndrome of sepsis. In resource-poor settings where many healthcare workers have minimal training and limited laboratory diagnostic capacity, syndromic identification represents a crucial method to triage sick children appropriately. Current guidelines aimed at identifying “danger signs” in children, such as the Integrated Management of Childhood Illness and Emergency Triage Assessment and Treatment manuals published by WHO, require healthcare workers to screen for signs of distress such as chest in-drawing and weak and fast pulses [9–10]. As such guidelines were largely developed from expert opinion, the predictive ability of each sign and symptom used remains to be evaluated in a systematic manner. The need for a sensitive and predictive screening tool is paramount; delayed recognition or ambiguity in prediction can be detrimental as early action is necessary for improved outcomes in the treatment of sepsis. A short delay in antibiotic administration can significantly increase mortality in patients with sepsis [11, 12].

Cognizant of the importance of a triage tool, we endeavored to develop a triage tool that can rapidly and reliably be used by low-skilled frontline healthcare workers to identify the children at risk of sepsis without the need for extensive memorization or training. As a first step we conducted a systematic review to generate a list of all possible predictors. We then used a modified Delphi process to build consensus on clinical signs, symptoms and vital signs that would be helpful to identify children at risk at the time of presentation. The output of this study will be a set of potential predictors based on expert consensus opinion, from both resource-rich and resource-limited settings, of their predictive potential, measurement reliability, and level of resources required to collect the predictor. These selected predictors will then be tested and reduced further in the next stage using data from a prospective cohort of children. With at least 10 outcomes per predictor, this typically requires a sample that included 200–300 outcomes. We will then derive a prediction model that can be integrated into a mobile device (e.g., smartphone, tablet, or standalone medical device) to screen all children at the time of presentation. The final objective of this multi-study project is to enable a low-skilled frontline healthcare worker to utilize a data-driven triage tool to identify children with sepsis or at risk of developing sepsis at the time of presentation to the healthcare facility so that resources can be allocated as quickly and as appropriately as possible.

## Methods

Ethics approval was obtained from the Child and Women’s Research Ethics Board at the University of British Columbia (H17-01893). Voluntary completion of the questionnaire implied consent. The participants' responses were received and analyzed anonymously.

### Systematic review

To generate a preliminary list of potential predictor variables of sepsis in children in resource-limited settings, a systematic search was conducted in June 2017 using the Ovid MEDLINE database. The search strategy included search terms such as “child”, “sepsis”, “triage”, “prediction”, “warning score”, “risk factor”, and “resource-limited settings”. The complete search strategy is available in S1 Appendix.

Studies were included if they represented a population of either community-based or facility-based children under 5 years of age, were conducted in a resource limited setting, and measured clinical signs and/or symptoms (including laboratory parameters) that were assessed against any outcome of disease severity or death. Pertinent signs or symptoms (e.g., predictor variables) used in the studies were abstracted to generate a master list. A potential predictor variable was identified if it was a sign or symptom, past medical history, and point-of-care testing available in resource-limited settings that enabled the identification of a sick child at presentation to a community healthcare worker or to a healthcare facility. The reference list of each manuscript was reviewed to identify potential studies for additional variables. Global guidance documents published by WHO including Integrated Management of Childhood Illness (IMCI), Integrated Community Case Management (ICCM), and Emergency Triage and Assessment (ETAT), as well as regional reference documents such as the South African Triage Scale (SATS) were also reviewed to abstract the danger and emergency signs identified by the technical advisory organizations [9–10,13–14].

The master list was created using Microsoft Excel to keep track of each predictor variable and the frequency of repetition in other studies as a presumed indicator of its’ predictive potential and relative commonality. Each variable was also classified as either a binary variable (responses are “yes” or “no”) versus continuous (numerical values, e.g., respiratory rate) and organized into categories such as “patient characteristics”, “vital signs”, “infection”, and “neurological signs”. The master list is available for reference in S2 Appendix.

### Modified Delphi process

#### Round 1

The first round of the Delphi process was initiated in November 2017 and completed by December 2017. An a priori decision was made by the research team at this time to set the final number of predictor variables to 45, based on the amount of training and resources that may be realistically collected in the development of the triage tool. Predictor variables generated from the literature review were suggested to participants to determine their opinion as to its value as a predictor of sepsis. If the participant responded “yes”, they were prompted to evaluate the variable according to three domains: predictive potential, measurement reliability, and the level of training and/or resources required to measure and collect the variable. These domains were adapted from our previous study in which we used a similar Delphi process to select candidate predictor variables for post-discharge mortality in children under 5 years of age [15]. Each domain consisted of 4 response choices: high, moderate, minimal, or not applicable. There was also an option for the participant to make comments regarding each variable. Participants were also encouraged to suggest additional variables and to evaluate these according to the three domains outlined. Each round was given a two-week deadline for completion. Reminders were sent at one week and two days before the deadline.

For analysis, each response option was assigned a number between 0 and 3 (0 = not applicable) based on the strength of the response. The sum for each domain for each variable was tabulated to calculate a weighted effect to help determine the selection threshold. The weighted effect was calculated by doubling the weight of the value for predictive potential, adding the value of measurement reliability and subtracting the value of level of resources and/or training required. The overall mean and median were calculated for the three domains as a reference. A threshold was chosen based on the desired number of predictors. Variables that scored above the threshold were included in the final set of predictor variables. If a close grouping was obtained at the threshold, predictors below the threshold were also included. Predictors that were below the threshold were carefully reviewed by the research team and a subset was included in Round 2 for re-evaluation. Any additional variables proposed by participants were also selected to be evaluated in Round 2 if they were considered clinically distinct from the variables already assessed in Round 1.

#### Round 2

The second round was conducted from January to February 2018 and it consisted of re-evaluating selected variables from Round 1 as well as newly suggested variables according to the same three domains. Participants were provided the average response responses to the three domains as well as their previously selected response. A similar threshold procedure was used for Round 2.

#### Participants

Participants were recruited to include as wide a range of expertise as possible. All participants are involved in the care of critically ill children and/or active in research in areas including: (1) pediatrics, (2) sepsis, (3) infectious diseases, (4) microbiology/laboratory medicine, (5) international health, (6) epidemiology, (7) social sciences, (8), neonatology, and (9) obstetrics. Invited participants were based in both resource-limited and high-resource settings. Participants were contacted through an email invitation explaining the objective of the study and inviting them to participate in both rounds of the Delphi process. Our target sample size of expert contributors was 20.

#### Definitions of the variables–SNOMED CT

To standardize the meaning of common clinical terminology used in the questionnaire, definitions were abstracted from SNOMED CT, a comprehensive multilingual clinical terminology database used for electronic health records globally. If studies had defined the variable themselves, these were compared with the standard SNOMEDCT definitions for any discrepancies. If a specific variable was not found within the SNOMED CT database, a definition was generated by the research team based on clinical and research experience. The data dictionary used in Round 1 is available for reference in S3 Appendix.

#### Development of the questionnaire–REDCap

The questionnaire was developed using the secure web-based application, Research Electronic Data Capture “REDCap”. [16] Each participant could return to the questionnaire if they were not able to complete it a single sitting. Participant’s own response from Round 1 was available in Round 2 along with a mean response from the other participants. Questionnaires used in both rounds are available for reference in S4 and S5 Appendices.

## Results

### Systematic review

The systematic search yielded 176 manuscripts. These were reviewed as abstracts and, when necessary, in full text. References of articles were also screened for potentially eligible manuscripts. This process identified 12 manuscripts that were used in this study, along with 3 publications from WHO and the South African Triage Scale developed by the Emergency Medicine Society of South Africa. The review generated 153 variables across the categories of patient characteristics, vital signs, and signs and symptoms by organ systems. Variables assessing trauma and surgical needs were also included in this initial list, as were variables that required laboratory testing such as hemoglobin and blood smear for malaria. The master list of variables was evaluated to determine if there were variables that represented the same clinical concept and could potentially be combined. For example, variables that were abstracted from different studies but were clinically identical, such as a binary variable of a temperature of greater than or equal to 37°C compared to a continuous variable of temperature, the latter was selected to be evaluated in the current study. The availability of diagnostic tools and timing for results at the point of triage were also taken into consideration when evaluating the utility of each variable. After this initial evaluation, a total of 72 predictor variables (Table 1) were selected to be evaluated in Round 1 of the Delphi process.

**Table 1:** Sums of the responses to the domains of all variables evaluated in Rounds 1 and 2.

	Responses in Round 1			Responses in Round 2			
Initial Variables	Predictability	Reliability	Resources	Predictability	Reliability	Resources	Outcome
**Patient Characteristics**							
***Accepted***							
Age	64	69	27				Y
Gender	44	74	23				Y
Duration of illness/sign/symptom	62	52	38				Y
Time since last hospitalization	46	47	35				Y
Urgent referral status	58	58	39				Y
Child HIV status	65	58	49				Y
**Anthropometric Data**							
***Accepted***							
Weight	64	58	43				Y
Middle upper arm circumference	65	61	43				Y
***Rejected***							
Length	36	33	31				N
**Vital Signs**							
***Accepted***							
Temperature	61	60	42				Y
Heart rate	58	56	40				Y
Respiratory rate	65	52	48				Y
Oxygen saturation	67	55	54				Y
***Re-evaluated***							
Systolic blood pressure	50	37	48	61	42	52	Y
**Airway and Breathing**							
***Accepted***							
Apnea (observed and/or reported)	59	45	42				Y
Difficulty breathing (reported)	45	40	33				Y
Difficulty breathing (observed)	65	53	51				Y
Chest in-drawing	62	53	54				Y
Grunting	54	49	44				Y
Increased respiratory effort	57	48	46				Y
***Re-evaluated***							
Central cyanosis	52	38	45	67	43	47	Y
Stridor	43	40	38	45	46	46	N
Nasal flaring	40	34	28	46	45	42	Y
Head bobbing/nodding	41	37	35	44	41	44	N
***Rejected***							
Wheezing	37	39	38				N
Obstructed breathing	40	31	38				N
Fast breathing (reported)	33	30	23				N
**Circulation**							
***Accepted***							
Capillary refill time	62	47	49				Y
Skin cold (cold peripheries)	53	46	38				Y
Weak and fast pulse	64	49	56				Y
Pallor–palmar, oral, conjunctival	51	44	39				Y
**Neurological**							
***Accepted***							
Irritability/restlessness	47	43	35				Y
Alert/Voice/Pain/Unresponsiveness	71	58	51				Y
Convulsions (reported; history of)	47	41	34				Y
Convulsing now, actively	53	55	36				Y
Not feeding well (reported)	46	37	28				Y
Not being able to drink or feed anything	60	54	31				Y
***Re***-***evaluated***							
Bulging fontanelles	50	43	46	64	51	44	Y
Neck pain/stiffness	48	38	43	57	45	46	Y
Reduced spontaneous movements/to stimulus	42	37	35	51	43	44	Y
***Rejected***							
Inconsolability	32	32	27				N
Ease of wakening	26	20	20				N
Lethargy	40	33	29				N
Stiff limbs	34	30	33				N
Hypotonia	43	34	41				N
**Dehydration**							
***Accepted***							
Reduced urine production	52	35	39				Y
***Re***-***evaluated***							
Skin turgor	48	38	45	46	41	47	N
Sunken eyes	41	38	37	47	44	43	Y
***Rejected***							
Depressed fontanelles	36	32	31				N
No tears when crying	28	26	23				N
Dry oral mucosa	25	24	24				N
**Infection**							
***Accepted***							
Fever	52	53	29				Y
***Re-evaluated***							
Cough	33	40	20	30	48	26	N
***Rejected***							
Runny nose	17	21	11				N
Rash	31	32	24				N
Ear pain	21	19	16				N
Ear discharge	25	25	17				N
Tender swelling behind ear	30	27	25				N
Purulent drainage from eyes	21	24	19				N
Conjunctivitis	17	17	15				N
Skin pustules	34	35	26				N
**GI/GU**							
***Accepted***							
Diarrhea	44	45	29				Y
Blood in stool (dysentery)	52	47	34				Y
Vomiting	41	48	26				Y
***Rejected***							
Abdominal pain	25	23	18				N
Foul-smelling urine	27	21	20				N
**Malnutrition**							
***Accepted***							
Swelling of both feet (peripheral edema)	48	43	37				Y
Visible severe wasting (marasmus)	58	51	38				Y
***Re-evaluated***							
Oral thrush	41	42	38	52	46	46	Y
Abdominal distension	40	38	39				N
**General**							
***Rejected***							
Change in level of activity	44	39	35				N
Change in crying	29	28	24				N
**New Variables for Round 2**							
Number of previous hospital admissions				53	58	29	Y
Jaundice				45	44	39	Y
Significant blood loss				53	40	52	N
Severe pain				45	41	48	N

The results for the three domains: “predictive potential”, “measurement reliability”, and “level of training and/or resources required” for each variable from the two-round modified Delphi process separated by categories. A total of 72 variables were evaluated in Round 1 and 4 new variables were evaluated in Round 2. Variables that were accepted in the final set of predictors are denoted with “Y” in the final column; variables that were rejected are denoted with “N”.

### Round 1

**Fig 1:**
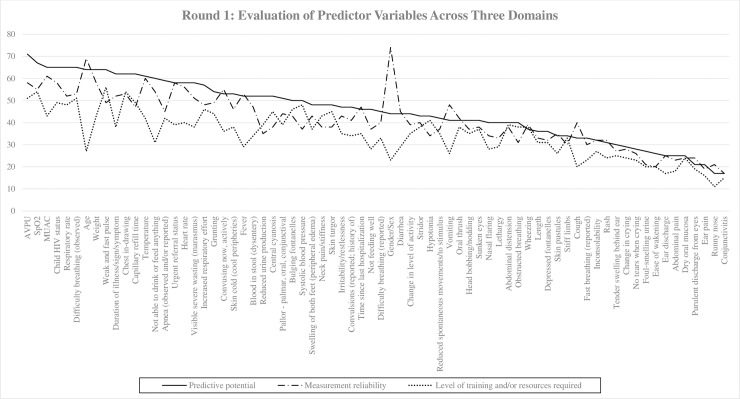
The trends of the sum of responses to each domain for all 72 variables evaluated in Round 1.

Twenty-six experts from high-resource and resource-limited settings participated in Round 1 of the modified Delphi process; these included physicians and researchers affiliated with teaching hospitals and universities with expertise in pediatrics, critical care, sepsis, and international health (Table 2). Participants were based in Uganda, Ghana, Kenya, Nigeria, Malawi, India, South Africa, Canada, and United States. Disciplines self-reported by experts include critical care, intensive care, and academic research in clinical and health services. Seventy-two variables across 11 categories were evaluated in Round 1 (Table 1). There was a consistent response between the domains (Fig 1; Table 1).

**Table 2:** Summary demographics of expert contributors (N = 26).

**Primary Affiliation**	**N**
Teaching Hospital	22
General/Community Hospital	4
Specialized Hospital	3
Outpatient Clinic	2
University	9
Other:	
Research Institute	3
Tertiary Hospital	3
Children’s Hospital	2
Doctors Without Border	1
**Role at the Institution**	**N**
Physician	21
Clinician	1
Clinician Scientist	9
Researcher	12
Hospital Administration	5
Nurse	2
Other:	
Clinical Instructor/Professor	3
**Area of Expertise**	**N**
Pediatrics	26
Sepsis	12
Infectious Disease	8
International Health	13
Epidemiology	5
Social Sciences	1
Neonatology	3
Other:	
Health Systems	2
Critical Care/Intensive Care	12
Global Health, Child Survival	1
Informatics	1

#### Proposed new variables

Twenty-five new variables were proposed by participants from Round 1. Following discussion, we excluded suggested variables such as “when was the last time the child was well” since they were already captured by variables evaluated in Round 1. Other variables such as “point of care malaria rapid diagnostic test” and “blood sugar levels” were excluded since these involved invasive tests and were likely not realistic to be performed at triage by a low skilled health worker. Similarly, suggestions that pertained to more detailed physical examinations such as “splenomegaly” or “regression of achieved developmental milestones” were excluded due to the complex training required. Four new variables were included in Round 2 for evaluation. These are the number of previous hospital admissions, jaundice, significant blood loss, and severe pain.

### Round 2

**Fig 2:**
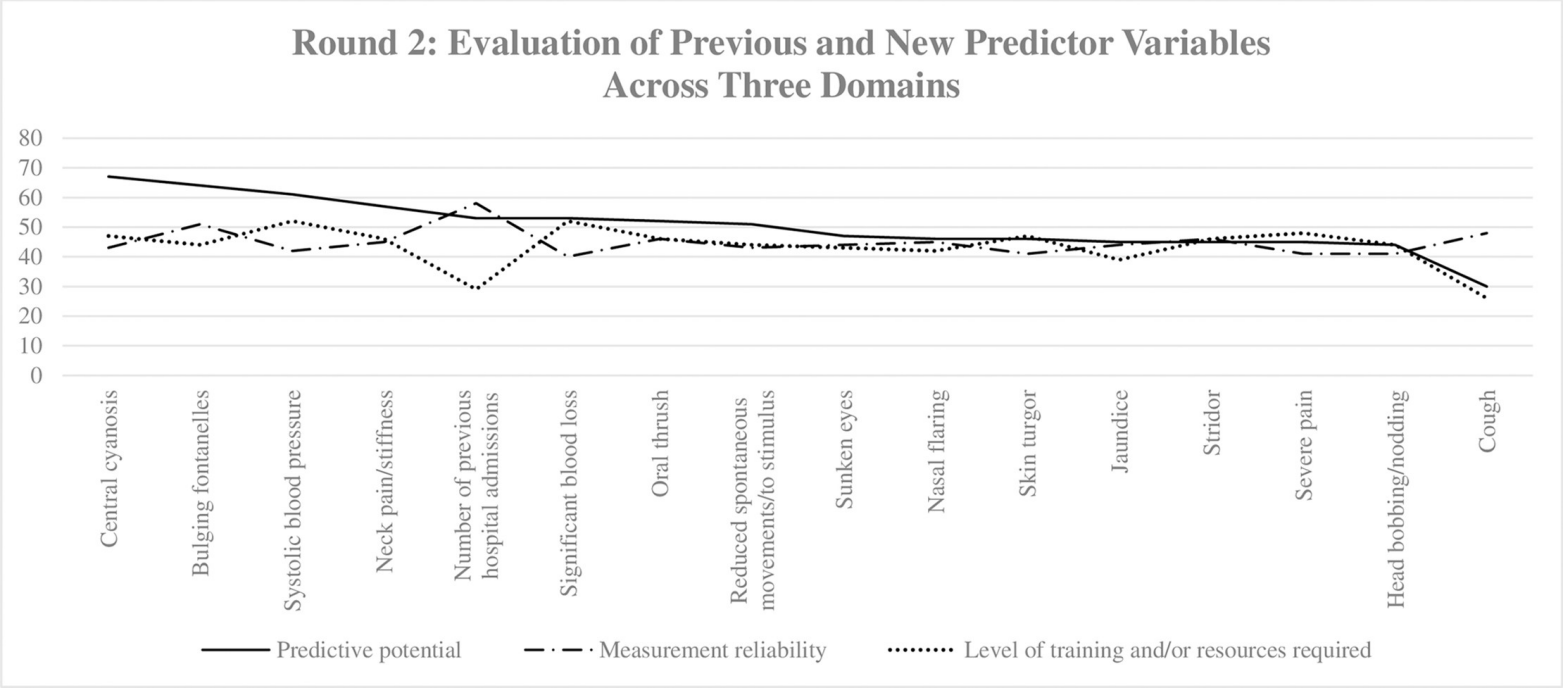
The trends of the sum of responses to each domain for all 16 variables evaluated in Round 2.

Twenty-five expert contributors from Round 1 completed the questionnaire for Round 2. The responses were consistent between the domains for all 16 variables (Fig 2).

#### Domain 1: Predictive potential

The five highest scoring variables for predictive potential include central cyanosis, bulging fontanelles, systolic blood pressure, neck pain/stiffness and number of previous hospital admissions. Of note, “number of previous hospital admissions” was a newly suggested variable for Round 2; all five highest scoring variables were accepted in the final set of predictors. The five lowest scoring variables for this domain include cough, head bobbing/nodding, severe pain, stridor, and jaundice. Even though “jaundice” was among the lowest scoring variables, likely due to the rarity of its appearance in children older than 2 months of age, it was accepted into the final set of predictors because of the clinical significance it holds when it is identified in newborns.

#### Domain 2: Measurement reliability

The five highest scoring variables for measurement reliability include number of previous hospital admissions, bulging fontanelles, cough, oral thrush, and stridor. The five lowest scoring variables for this domain include significant blood loss, head bobbing/nodding, severe pain, reduced skin turgor, and systolic blood pressure. Comments from participants for the lowest scoring variables included concerns with quantification of blood loss, and pain which can affect the objectivity of the assessment during triage.

#### Domain 3: Level of training and/or resources required

The five highest scoring variables for level of training and/or resources required include systolic blood pressure, significant blood loss, severe pain, central cyanosis, and reduced skin turgor. The five lowest scoring variables for this domain include cough, number of previous hospital admissions, jaundice, nasal flaring, and sunken eyes. Systolic blood pressure remained in the highest scoring variables for the level of resources required as it did for Round 1, indicating that even though the predictive potential of systolic blood pressure is high, tools such as sphygmomanometers and stethoscopes (or automated measurement devices) are likely to be lacking in resource-limited settings, as well as the amount of training a healthcare worker must undergo to obtain an accurate reading may not be feasible.

## Discussion

The systematic review identified 72 potential predictors that were reduced to 45 using a modified two-round Delphi process. (Table 3) These predictors will be used to collect data in a large cohort of patients which will then be used to create the initial prediction model.

**Table 3:** Final set of predictor variables (N = 45).

Patient Characteristics	Circulation
Age	Capillary refill time
Gender	Weak and fast pulse
Duration of illness/sign/symptom	Skin cold (cold peripheries)
Urgent referral status	Pallor–palmar, oral, conjunctival
Time since last hospitalization	**Neurological**
Number of previous hospital admissions	Alert/Voice/Pain/Unresponsiveness
Child HIV status	Not being able to feed or drink anything
**Anthropometric Data**	Not feeding well (reported)
Weight	Irritability/restlessness
Middle upper arm circumference	Convulsing now, actively
**Vitals**	Convulsions (reported; history of)
Heart Rate	Bulging fontanelles
Respiratory Rate	Neck pain/stiffness
Temperature	Reduced spontaneous movements/to stimulus
Oxygen saturation	**GI/GU**
Systolic blood pressure	Diarrhea
**Respiratory**	Blood in stool (dysentery)
Chest in-drawing	Vomiting
Apnea (observed and/or reported)	Jaundice
Increased respiratory effort	**Malnutrition**
Central cyanosis	Visible severe wasting (marasmus)
Difficulty breathing (observed)	Swelling in both feet (peripheral edema)
Difficulty breathing (reported)	Oral thrush
Nasal flaring	**Dehydration**
Grunting	Reduced urine production
**Infection**	Sunken eyes
Fever	

We chose the Delphi process to achieve consensus among a panel of experts on a defined issue using iterations of questionnaires and aggregating the responses to provide feedback to the participants after each completed round. This method of generating consensus is widely applied in diverse fields such as program planning and needs assessments, and in the healthcare sector [17–18]. An advantage to using this method is the facilitation of consensus-building online and the elimination of the need to have face-to-face interactions, which significantly enabled the participation of subject matter experts from disparate locations worldwide. We chose to conduct a modified version of the Delphi process whereby a literature review was performed to generate a list of preliminary predictor variables which formed the basis of the questionnaire for Round 1. This was done to systematically identify all the possible variables that may be required to be evaluated and to eliminate further time needed of the participants to generate an extensive list de novo. We then invited participants to propose additional variables. This approach was an attempt to identify the most exhaustive list of clinical predictors possible, identified from the literature as well as from the clinical experience of participants.

The responses from the 26 participants from both resource-rich and resource-limited settings were crucial in allowing the condensation of the initial list of 72 predictor variables to 45 predictors after consideration of the predictive potential, measurement reliability, and the level of resources and/or training required for each variable. In addition to the numerical responses, the ability of the participants to give comments provided insights as to the feasibility of collecting data on certain variables when considering our overarching objective–to allow the identification of a sick child using a triage tool operated by a low skilled healthcare worker.

Future steps will be to determine collinearity between independent variables using the data collected from the large cohort. This will be important to help reduce the number of questions that must be asked at triage to ensure appropriate care delivery as soon as possible. Currently, for example, the ETAT and SATS guidelines both require identifying and inquiring about over 50 signs and symptoms. In the next phase, the optimum predictors will be selected based on outcomes such as mortality, need for referral of admission and biomarkers using data from a prospective cohort of sick children presenting to a healthcare facility. It is anticipated that the prediction model derived from the prospective data will be in the order of 10–15 variables. The development and use of a simple tool built on the basis of signs and symptoms with moderate to strong predictive potentials, will ideally facilitate the triage process and expedite the care for a sick child with evolving sepsis.

### Limitations

This study is subjected to several limitations. The first limitation is including variables, such as jaundice in a child older than 2 months old, that may be rare events but are unlikely to be useful in a generalizable prediction model. These variables may, however, be critical when they are present; thus, we included these variables for consideration [19]. Similarly, certain clinical signs or symptoms are context specific. For example, they may be more common in certain age groups, such as jaundice in a newborn. As we have chosen to include all predictors for children under 5 years of age, we elected to include age-limited variables as the presence of these signs may be crucial in identification of a sick child in that age group. We have not stratified and studied infants and newborns separately although they are likely to manifest different disease characteristics and likely different pathobiology. A review of the newborn and infant as a distinct sub-population will be required. As such, the results of this review should be applied with caution in the neonatal population. Expert opinions may have significant limitations, as this may be impacted by training, exposure, and expertise. To mitigate bias, our target sample size was set at 20 which we surpassed with 26 experts contributing in Round 1 and 25 experts contributing in Round 2. Lastly, although we are aiming to develop a triage tool, we primarily focused on clinical predictors for sepsis in this study. Conditions that are not related to sepsis such as trauma, but equally important in terms of critical care and management upon presentation to the healthcare facility, were not included. We anticipate that these children would be rapidly identified at presentation and would not need to undergo triage before being assessed and managed. We have focused on predictors of sepsis as it is a major cause of mortality in children under 5 years of age in resource-limited settings.

## Conclusion

The modified Delphi process enabled the distillation of an initial list of 72 predictor variables, identified through a systematic review, for identification of a sick child under 5, to a final list of 45 predictor variables. This was achieved using feedback from 26 expert contributors with different backgrounds from around the world. The final list of candidate predictor variables will be used to derive a prediction model that may be used to triage sick children at the time of presentation.

## Supporting information

S1 Appendix Search strategy for literature review. (DOCX)

S2 Appendix Master list of all potential variables from literature review. (XLSX)

S3 Appendix Data dictionary for variables evaluated in Round 1. (DOCX)

S4 Appendix Questionnaire for Round 1 of the modified Delphi process. (PDF)

S5 Appendix Questionnaire for Round 2 of the modified Delphi process. (PDF)

## References

[1] Children: reducing mortality. World Health Organization [Internet]. 2017 Oct [cited 2018 Jul 02]. Available from: http://www.who.int/news-room/fact-sheets/detail/children-reducing-mortality

[2] Global Burden of Disease Collaborative Network. Global Burden of Disease Study (2016) (GBD 2016) Cause-Specific Mortality 1980–2016. Seattle, United States: Institute for Health Metrics and Evaluation (IHME), 2017.

[3] Kissoon N and Uyeki TM. Sepsis and the Global Burden of Disease in Children. JAMA Pediatrics 2016 2; 170(2): 107–108. 10.1001/jamapediatrics.2015.3241 26661465 PMC6636321

[4] Fleischmann-Struzek C, Goldfarb DM, Schlattmann P, Schlapbach LJ, Reinhart K, and Kissoon N. The global burden of paediatric and neonatal sepsis: a systemic review. Lancet Respir Med 2018 3; 6: 223–230. 10.1016/S2213-2600(18)30063-8 29508706

[5] Rudd KE, Kissoon N, Limmathurotsakul D, Bory S, Mutahunga B, Seymour CW et al The global burden of sepsis: barriers and potential solutions. Critical Care 2018 9; 22(1):232 10.1186/s13054-018-2157-z 30243300 PMC6151187

[6] WHA Adopts Resolution on Sepsis. Global Sepsis Alliance [Internet]. 2017 May [cited 2018 Jul 02]. Available from: https://www.global-sepsis-alliance.org/news/2017/5/26/wha-adopts-resolution-on-sepsis

[7] Reinhart K, Daniels R, Kissoon N, Machado FR., Schachter RD, and Finfer S. Recognizing Sepsis as a Global Health Priority–A WHO Resolution. N Engl J Med 2017 8; 377: 414–417. 10.1056/NEJMp1707170 28658587

[8] Kissoon N, Reinhart K, Daniels R, Machado MFR, Schachter RD, and Finfer S. Sepsis in Children: Global Implications of the World Health Assembly Resolution on Sepsis. Pediatric Crit Care Med 2017 12; 18(12): e625–e627. 10.1097/PCC.0000000000001340 28914721

[9] IMCI chart booklet. World Health Organization [Internet]. 2014 Mar [cited 2018 Jul 02]. Available from: http://www.who.int/maternal_child_adolescent/documents/IMCI_chartbooklet/en/

[10] Emergency Triage Assessment and Treatment (ETAT) course. World Health Organization [Internet]. 2005 [cited 2018 Jul 02]. Available from: http://www.who.int/maternal_child_adolescent/documents/9241546875/en/

[11] Kumar A, Roberts D, Wood KE, Light B, Parrillo JE, Sharma S et al Duration of hypotension before initiation of effective antimicrobial therapy is the critical determinant of survival in human septic shock. Crit Care Med. 2006 6; 34(6): 1589–1596. 10.1097/01.CCM.0000217961.75225.E9 16625125

[12] Weiss SL, Fitzgerald JC, Balamuth F, Alpern ER, Lavelle J, Chilutti M et al Delayed Antimicrobial Therapy Increases Mortality and Organ Dysfunction Duration in Pediatric Sepsis. Critical Care Medicine. 2014 11; 42 (11): 2409–2417. 10.1097/CCM.0000000000000509 25148597 PMC4213742

[13] Caring for the sick child. World Health Organization [Internet]. 2011 [cited 2018 Jul 02]. Available from: http://www.who.int/maternal_child_adolescent/documents/caring-for-the-sick-child/en/

[14] The South African Triage Scale (SATS). Emergency Medicine Society of South Africa. 2012 [cited 2018 Jul 02]. Available from: https://emssa.org.za/sats/

[15] Wiens MO, Kissoon N, Kumbakumba E, Singer J, Moschovis PP, Ansermino J, et al Selecting candidate predictor variables for the modelling of post-discharge mortality from sepsis: a protocol development project. Afr Health Sci. 2016 3; 16(1):162–169. 10.4314/ahs.v16i1.22 27358628 PMC4915422

[16] Harris PA, Taylor R, Thielke R, Payne J, Gonzalez N, and Conde JG, Research electronic data capture (REDCap)–A metadata-driven methodology and workflow process for providing translational research informatics support, J Biomed Inform. 2009 4; 42(2):377–81 10.1016/j.jbi.2008.08.010 18929686 PMC2700030

[17] Hsu CC and Sandford BA. The Delphi technique: making sense of consensus. Practical Assessment, Research & Evaluation. 2007; 12:1–8.

[18] Eubank BH, Mohtadi NG, Lafave MR, Wiley JP, Bois AJ, Boorman RS, et al Using the modified Delphi method to establish clinical consensus for the diagnosis and treatment of patients with rotator cuff pathology. BMC Med Res Methodol. 2016 May; 16:56 10.1186/s12874-016-0165-8 27206853 PMC4875724

[19] Pashankar D and Schreiber RA. Jaundice in Older Children and Adolescents. Pediatrics in Review. 2001 7; 22(7):219–226. 11435623 10.1542/pir.22-7-219

