# Supplementary material for: Determining predictors of sepsis at triage among children under 5 years of age in resource-limited settings: A modified Delphi process
Source: PLoS One. 2019 Jan 28;14(1):e0211274. doi: 10.1371/journal.pone.0211274 (PMC6349330; doi:10.1371/journal.pone.0211274)
Supplement: S1 Appendix — (DOCX) [file pone.0211274.s001.docx]

**S1 Appendix – Search Strategy for Literature Review**

1. exp Sepsis/
2. *sepsis.mp.*
3. 1 or 2
4. exp Triage/
5. prediction.mp.
6. warning score.mp.
7. risk factor*.mp.
8. or/4-7
9. 3 and 8
10. global health/
11. developing countr*.mp.
12. resource limited settings*.mp.
13. or/10-12
14. 9 and 13
15. exp child/ or exp infant/
16. 14 and 15
