# Supplementary material for: Determining predictors of sepsis at triage among children under 5 years of age in resource-limited settings: A modified Delphi process
Source: PLoS One. 2019 Jan 28;14(1):e0211274. doi: 10.1371/journal.pone.0211274 (PMC6349330; doi:10.1371/journal.pone.0211274)
Supplement: S3 Appendix — (DOCX) [file pone.0211274.s003.docx]

**S3 Appendix – Data Dictionary for Variables Evaluated in Round 1**

| **Predictor Variable** | **Definition** |
| --- | --- |
| **Patient Characteristics** | |
| Age | Current chronological age. |
| Gender/Sex | Patient sex. |
| Duration of illness/sign/symptom | Duration of illness. |
| Time since last hospitalization | Time elapsed since the patient’s previous hospitalization, if any. |
| Urgent referral status | The child is referred on an urgent basis from a primary healthcare facility. |
| Child HIV status | The patient’s HIV status at time of presentation (positive, negative, unknown). |
| **Anthropometric** **Data** | |
| Weight | Body weight. |
| Length | Length of body. |
| Middle upper arm circumference | A circumferential measurement of the largest part of the upper arm. |
| **Vital Signs** | |
| Temperature | No definition provided. |
| Heart rate | No definition provided. |
| Respiratory rate | No definition provided. |
| Oxygen saturation (SpO2) | No definition provided. |
| Systolic blood pressure | No definition provided. |
| **Airway and Breathing** | |
| Apnea (observed and/or reported) | Transient cessation of breathing. |
| Difficulty breathing (reported) | Difficult or laboured breathing, reported by the caregiver. |
| Difficulty breathing (observed) | Difficult or laboured breathing, observed by the healthcare worker. |
| Central cyanosis | A blue discoloration that occurs when there is a decrease in oxygen saturation in the arterial blood, usually with an SpO2 of <75%. |
| Chest in-drawing | Lower chest wall retraction when the child breathes in. |
| Obstructed breathing | Can be due to blockage by the tongue, a foreign body, a swelling around the upper airway or severe croup. |
| Nasal flaring | Widening of the nostrils upon inhalation as a manifestation of respiratory distress. |
| Grunting | A short noise when breathing out with increased effort in young infants. |
| Head bobbing/nodding | Head is bobbing up and down due to usage of accessory muscles for respiration. |
| Stridor | High-pitched sound on inspiration produced by obstruction of the upper airway. |
| Wheezing | A high-pitched whistling sound associated with laboured breathing. |
| Increased respiratory effort | Increased respiratory rate and use of accessory muscles to breathe. |
| Fast breathing (reported) | Caregiver reports the child as breathing very fast. |
| **Circulation** | |
| Capillary refill time | The time it takes from the release of the blanching pressure applied to the nailbed of the thumb or big toe to complete return of the pink colour. |
| Skin cold (cold peripheries) | Assessed by having the healthcare worker feel the child’s hand. |
| Weak and fast pulse | A weak and fast pulse felt at any of the following locations: radial, brachial, femoral, carotid. |
| Pallor – palmar, oral, conjunctival | An unusual or extreme paleness, state of decreased skin or mucosal coloration observed at the palms, oral cavity, and conjunctiva. |
| **Dehydration** | |
| Skin turgor | Pinch the skin of the abdomen and see whether the skin goes back very slowly (longer than 2 seconds). |
| Sunken eyes | Look at the child’s eyes to determine if they appear unusually sunken in their sockets. |
| Depressed fontanelles | Sunken fontanelles |
| No tears when crying | Little to not tears when the child is crying. |
| Reduced urine production | Less urine than usually produced based on diaper changes or observed micturition. |
| Dry oral mucosa | Appearance of tissue desiccation in the oral cavity. |
| **Neurological** | |
| Irritability/restlessness | A child who is conscious but cries constantly and will not settle. |
| Inconsolability | A child who is conscious but cries constantly and will not settle. |
| Alert/ voice/ pain/ unresponsive (AVPU) | A child who is determined to be alert, responsive to voice, responsive to pain, or is unresponsive at all. |
| Ease of wakening | The child is difficult to wake, reported by the caregiver. |
| Lethargy | A child who is not alert but responds to voice is lethargic. |
| Convulsions (reported; history of) | Sudden involuntary skeletal muscular contractions, reported by the caregiver as having occurred. |
| Convulsing now, actively | The child is currently experiencing sudden, involuntary skeletal muscular contractions. |
| Stiff limbs | A display of muscle rigidity in the child’s limbs. |
| Hypotonia | Decreased tone of the skeletal muscles marked by a diminished resistance to passive stretching. |
| Reduced spontaneous movements/ to stimulus | Child exhibits reduced spontaneous movements and does not respond with appropriate movements to stimuli. |
| Neck pain/stiffness | Limited mobility of the neck, usually accompanied by pain. |
| Bulging fontanelles | Fontanelles appear elevated or full. |
| Not feeding well | Ask the caregiver if the child is having difficulty in drinking or feeding. |
| Not being able to drink or feed anything | A child is not able to drink or feed if they are too weak to suckle or swallow when offered a drink or breastmilk. |
| **Infection** | |
| Fever | Elevated body temperature assessed by caregiver (qualitative or quantitative). |
| Cough | Forced exhalation |
| Runny nose | Increased nasal secretions. |
| Rash | New diffuse macular or popular rash of the skin. |
| Ear pain | Ear pain reported by the caregiver. |
| Ear discharge | Discharge or drainage of fluids from the ear. |
| Tender swelling behind ear | Observed tenderness or swelling behind the ear: a sign of mastoiditis. |
| Purulent drainage from eyes. | Liquid exudate from the eye. |
| Conjunctivitis | Inflammation of the mucous membrane that lines the inner surface of the eyelids. |
| Skin pustules | A circumscribed and elevated skin lesion filled with purulent material. |
| **GI & GU** | |
| Diarrhea | Passage of loose, unformed stools. |
| Blood in stool (dysentery) | Appearance of blood in stools. |
| Vomiting | Vomiting everything; unable to keep anything down. |
| Abdominal pain | Child expresses sensation of discomfort, distress, or agony in the abdominal region. |
| Foul-smelling urine | Report or observation of abnormal odour of urine. |
| Malnutrition |  |
| Swelling of both feet (peripheral edema) | Observing of prolonged duration of impression in the skin following gentle compression. |
| Visible severe wasting (marasmus) | Evidence of severe loss of muscle and fat with prominent boney structures. |
| Abdominal distension | Visible distension of the abdomen. |
| Oral thrush | Ulcers or white patches in the mouth. |
| **General** | |
| Change in level of activity | Compared to baseline, reported by the caregiver |
| Change in crying | Compared to baseline, reported by the caregiver. |
