# Supplementary material for: Determining predictors of sepsis at triage among children under 5 years of age in resource-limited settings: A modified Delphi process
Source: PLoS One. 2019 Jan 28;14(1):e0211274. doi: 10.1371/journal.pone.0211274 (PMC6349330; doi:10.1371/journal.pone.0211274)
Supplement: S4 Appendix — (PDF) [file pone.0211274.s004.pdf]

Expert Source

Record ID ______________________________

What are your primary affiliations? Check all that apply.

- ☐ Teaching hospital
- ☐ General/community hospital
- ☐ Specialized hospital
- ☐ Outpatient clinic
- ☐ University
- ☐ Other

Please specify. ______________________________

What are your roles at this institution? Check all that apply.

- ☐ Physician
- ☐ Other clinician
- ☐ Basic scientist
- ☐ Clinical scientist
- ☐ Social scientist
- ☐ Hospital administration
- ☐ Nurse
- ☐ Other

Please specify. ______________________________

What is your area of expertise? Check all that apply.

- ☐ Pediatrics
- ☐ Sepsis
- ☐ Infectious Disease
- ☐ Microbiology/laboratory medicine
- ☐ International Health
- ☐ Epidemiology
- ☐ Social Sciences
- ☐ Neonatology
- ☐ Obstetrics

Do you have any other relevant areas of expertise? ______________________________

Patient Characteristics

Age

Should this variable be collected?
- ◯ Yes
- ◯ No

How strong is this variable as a predictor of severe illness in children?
- ◯ Strong
- ◯ Moderate
- ◯ Unlikely

Can this variable be reliably measured (i.e. inter- and intra-rater reliability)
- ◯ Highly reliable
- ◯ Moderately reliably
- ◯ Unreliably

How much training will be required to collect the variable?
- ◯ Extensive
- ◯ Moderate
- ◯ Minimal

How much will this variable overlap with other variables?
- ◯ Significant overlap
- ◯ Minimal overlap
- ◯ Not applicable

Which variables will this variable overlap with? ______________________________

How common is this variable abnormal?
- ◯ Frequently
- ◯ Less than half the time
- ◯ Rarely
- ◯ Not applicable

Less than 2 months of age

Should this variable be collected?
- ◯ Yes
- ◯ No

How strong is this variable as a predictor of severe illness in children?
- ◯ Strong
- ◯ Moderate
- ◯ Unlikely

Can this variable be reliably measured (i.e. inter- and intra-rater reliability)
- ◯ Highly reliable
- ◯ Moderately reliably
- ◯ Unreliably

How much training will be required to collect the variable?
- ◯ Extensive
- ◯ Moderate
- ◯ Minimal

How much will this variable overlap with other variables?
- ◯ Significant overlap
- ◯ Minimal overlap
- ◯ Not applicable

Which variables will this variable overlap with? ______________________________

How common is this variable abnormal?
- ◯ Frequently
- ◯ Less than half the time
- ◯ Rarely
- ◯ Not applicable

Gender/Sex

Should this variable be collected?
- ◯ Yes
- ◯ No

How strong is this variable as a predictor of severe illness in children?
- ◯ Strong
- ◯ Moderate
- ◯ Unlikely

Can this variable be reliably measured (i.e. inter- and intra-rater reliability)
- ◯ Highly reliable
- ◯ Moderately reliably
- ◯ Unreliably

How much training will be required to collect the variable?
- ◯ Extensive
- ◯ Moderate
- ◯ Minimal

How much will this variable overlap with other variables?
- ◯ Significant overlap
- ◯ Minimal overlap
- ◯ Not applicable

Which variables will this variable overlap with? ______________________________

How common is this variable abnormal?
- ◯ Frequently
- ◯ Less than half the time
- ◯ Rarely
- ◯ Not applicable

Duration of illness/sign/symptom

Should this variable be collected?
- ◯ Yes
- ◯ No

How strong is this variable as a predictor of severe illness in children?
- ◯ Strong
- ◯ Moderate
- ◯ Unlikely

Can this variable be reliably measured (i.e. inter- and intra-rater reliability)
- ◯ Highly reliable
- ◯ Moderately reliably
- ◯ Unreliably

How much training will be required to collect the variable?
- ◯ Extensive
- ◯ Moderate
- ◯ Minimal

How much will this variable overlap with other variables?
- ◯ Significant overlap
- ◯ Minimal overlap
- ◯ Not applicable

Which variables will this variable overlap with? ______________________________

How common is this variable abnormal?
- ◯ Frequently
- ◯ Less than half the time
- ◯ Rarely
- ◯ Not applicable

Time since last hospitalization

Should this variable be collected?
- ◯ Yes
- ◯ No

How strong is this variable as a predictor of severe illness in children?
- ◯ Strong
- ◯ Moderate
- ◯ Unlikely

Can this variable be reliably measured (i.e. inter- and intra-rater reliability)
- ◯ Highly reliable
- ◯ Moderately reliably
- ◯ Unreliably

How much training will be required to collect the variable?
- ◯ Extensive
- ◯ Moderate
- ◯ Minimal

How much will this variable overlap with other variables?
- ◯ Significant overlap
- ◯ Minimal overlap
- ◯ Not applicable

Which variables will this variable overlap with? ________________________________

How common is this variable abnormal?
- ◯ Frequently
- ◯ Less than half the time
- ◯ Rarely
- ◯ Not applicable

Urgent referral status

Should this variable be collected?
- ◯ Yes
- ◯ No

How strong is this variable as a predictor of severe illness in children?
- ◯ Strong
- ◯ Moderate
- ◯ Unlikely

Can this variable be reliably measured (i.e. inter- and intra-rater reliability)
- ◯ Highly reliable
- ◯ Moderately reliably
- ◯ Unreliably

How much training will be required to collect the variable?
- ◯ Extensive
- ◯ Moderate
- ◯ Minimal

How much will this variable overlap with other variables?
- ◯ Significant overlap
- ◯ Minimal overlap
- ◯ Not applicable

Which variables will this variable overlap with? ________________________________

How common is this variable abnormal?
- ◯ Frequently
- ◯ Less than half the time
- ◯ Rarely
- ◯ Not applicable

Child HIV Positive

Should this variable be collected?

- ◯ Yes
- ◯ No

How strong is this variable as a predictor of severe illness in children?

- ◯ Strong
- ◯ Moderate
- ◯ Unlikely

Can this variable be reliably measured (i.e. inter- and intra-rater reliability)

- ◯ Highly reliable
- ◯ Moderately reliably
- ◯ Unreliably

How much training will be required to collect the variable?

- ◯ Extensive
- ◯ Moderate
- ◯ Minimal

How much will this variable overlap with other variables?

- ◯ Significant overlap
- ◯ Minimal overlap
- ◯ Not applicable

Which variables will this variable overlap with? __________________________________

How common is this variable abnormal?

- ◯ Frequently
- ◯ Less than half the time
- ◯ Rarely
- ◯ Not applicable

Anthropometric Data

Weight

Should this variable be collected?
- ◯ Yes
- ◯ No

How strong is this variable as a predictor of severe illness in children?
- ◯ Strong
- ◯ Moderate
- ◯ Unlikely

Can this variable be reliably measured (i.e. inter- and intra-rater reliability)
- ◯ Highly reliable
- ◯ Moderately reliably
- ◯ Unreliably

How much training will be required to collect the variable?
- ◯ Extensive
- ◯ Moderate
- ◯ Minimal

How much will this variable overlap with other variables?
- ◯ Significant overlap
- ◯ Minimal overlap
- ◯ Not applicable

Which variables will this variable overlap with? ______________________________

How common is this variable abnormal?
- ◯ Frequently
- ◯ Less than half the time
- ◯ Rarely
- ◯ Not applicable

Length

Should this variable be collected?
- ◯ Yes
- ◯ No

How strong is this variable as a predictor of severe illness in children?
- ◯ Strong
- ◯ Moderate
- ◯ Unlikely

Can this variable be reliably measured (i.e. inter- and intra-rater reliability)
- ◯ Highly reliable
- ◯ Moderately reliably
- ◯ Unreliably

How much training will be required to collect the variable?
- ◯ Extensive
- ◯ Moderate
- ◯ Minimal

How much will this variable overlap with other variables?
- ◯ Significant overlap
- ◯ Minimal overlap
- ◯ Not applicable

Which variables will this variable overlap with? ______________________________

How common is this variable abnormal?
- ◯ Frequently
- ◯ Less than half the time
- ◯ Rarely
- ◯ Not applicable

MUAC (mm)

Should this variable be collected?	◯ Yes<br>◯ No
How strong is this variable as a predictor of severe illness in children?	◯ Strong<br>◯ Moderate<br>◯ Unlikely
Can this variable be reliably measured (i.e. inter- and intra-rater reliability)	◯ Highly reliable<br>◯ Moderately reliably<br>◯ Unreliably
How much training will be required to collect the variable?	◯ Extensive<br>◯ Moderate<br>◯ Minimal
How much will this variable overlap with other variables?	◯ Significant overlap<br>◯ Minimal overlap<br>◯ Not applicable
Which variables will this variable overlap with?	______________________
How common is this variable abnormal?	◯ Frequently<br>◯ Less than half the time<br>◯ Rarely<br>◯ Not applicable

Vitals

Temperature

Should this variable be collected?
- ◯ Yes
- ◯ No

How strong is this variable as a predictor of severe illness in children?
- ◯ Strong
- ◯ Moderate
- ◯ Unlikely

Can this variable be reliably measured (i.e. inter- and intra-rater reliability)
- ◯ Highly reliable
- ◯ Moderately reliably
- ◯ Unreliably

How much training will be required to collect the variable?
- ◯ Extensive
- ◯ Moderate
- ◯ Minimal

How much will this variable overlap with other variables?
- ◯ Significant overlap
- ◯ Minimal overlap
- ◯ Not applicable

Which variables will this variable overlap with? ______________________________

How common is this variable abnormal?
- ◯ Frequently
- ◯ Less than half the time
- ◯ Rarely
- ◯ Not applicable

Temperature < 35.5C

Should this variable be collected?
- ◯ Yes
- ◯ No

How strong is this variable as a predictor of severe illness in children?
- ◯ Strong
- ◯ Moderate
- ◯ Unlikely

Can this variable be reliably measured (i.e. inter- and intra-rater reliability)
- ◯ Highly reliable
- ◯ Moderately reliably
- ◯ Unreliably

How much training will be required to collect the variable?
- ◯ Extensive
- ◯ Moderate
- ◯ Minimal

How much will this variable overlap with other variables?
- ◯ Significant overlap
- ◯ Minimal overlap
- ◯ Not applicable

Which variables will this variable overlap with? ______________________________

How common is this variable abnormal?
- ◯ Frequently
- ◯ Less than half the time
- ◯ Rarely
- ◯ Not applicable

Temperature >/37.5C

Should this variable be collected?

- ◯ Yes
- ◯ No

How strong is this variable as a predictor of severe illness in children?

- ◯ Strong
- ◯ Moderate
- ◯ Unlikely

Can this variable be reliably measured (i.e. inter- and intra-rater reliability)

- ◯ Highly reliable
- ◯ Moderately reliably
- ◯ Unreliably

How much training will be required to collect the variable?

- ◯ Extensive
- ◯ Moderate
- ◯ Minimal

How much will this variable overlap with other variables?

- ◯ Significant overlap
- ◯ Minimal overlap
- ◯ Not applicable

Which variables will this variable overlap with? __________________________

How common is this variable abnormal?

- ◯ Frequently
- ◯ Less than half the time
- ◯ Rarely
- ◯ Not applicable

Heart Rate

Should this variable be collected?

- ◯ Yes
- ◯ No

How strong is this variable as a predictor of severe illness in children?

- ◯ Strong
- ◯ Moderate
- ◯ Unlikely

Can this variable be reliably measured (i.e. inter- and intra-rater reliability)

- ◯ Highly reliable
- ◯ Moderately reliably
- ◯ Unreliably

How much training will be required to collect the variable?

- ◯ Extensive
- ◯ Moderate
- ◯ Minimal

How much will this variable overlap with other variables?

- ◯ Significant overlap
- ◯ Minimal overlap
- ◯ Not applicable

Which variables will this variable overlap with? __________________________

How common is this variable abnormal?

- ◯ Frequently
- ◯ Less than half the time
- ◯ Rarely
- ◯ Not applicable

Respiratory Rate

Should this variable be collected?
- ◯ Yes
- ◯ No

How strong is this variable as a predictor of severe illness in children?
- ◯ Strong
- ◯ Moderate
- ◯ Unlikely

Can this variable be reliably measured (i.e. inter- and intra-rater reliability)
- ◯ Highly reliable
- ◯ Moderately reliably
- ◯ Unreliably

How much training will be required to collect the variable?
- ◯ Extensive
- ◯ Moderate
- ◯ Minimal

How much will this variable overlap with other variables?
- ◯ Significant overlap
- ◯ Minimal overlap
- ◯ Not applicable

Which variables will this variable overlap with? ______________________________

How common is this variable abnormal?
- ◯ Frequently
- ◯ Less than half the time
- ◯ Rarely
- ◯ Not applicable

Oxygen Saturation (SpO2)

Should this variable be collected?
- ◯ Yes
- ◯ No

How strong is this variable as a predictor of severe illness in children?
- ◯ Strong
- ◯ Moderate
- ◯ Unlikely

Can this variable be reliably measured (i.e. inter- and intra-rater reliability)
- ◯ Highly reliable
- ◯ Moderately reliably
- ◯ Unreliably

How much training will be required to collect the variable?
- ◯ Extensive
- ◯ Moderate
- ◯ Minimal

How much will this variable overlap with other variables?
- ◯ Significant overlap
- ◯ Minimal overlap
- ◯ Not applicable

Which variables will this variable overlap with? ______________________________

How common is this variable abnormal?
- ◯ Frequently
- ◯ Less than half the time
- ◯ Rarely
- ◯ Not applicable

Systolic Blood Pressure

Should this variable be collected?
- ◯ Yes
- ◯ No

How strong is this variable as a predictor of severe illness in children?
- ◯ Strong
- ◯ Moderate
- ◯ Unlikely

Can this variable be reliably measured (i.e. inter- and intra-rater reliability)
- ◯ Highly reliable
- ◯ Moderately reliably
- ◯ Unreliably

How much training will be required to collect the variable?
- ◯ Extensive
- ◯ Moderate
- ◯ Minimal

How much will this variable overlap with other variables?
- ◯ Significant overlap
- ◯ Minimal overlap
- ◯ Not applicable

Which variables will this variable overlap with? ______________________________

Should this variable be collected?
- ◯ Frequently
- ◯ Less than half the time
- ◯ Rarely
- ◯ Not applicable

Diastolic Blood Pressure

Should this variable be collected?
- ◯ Yes
- ◯ No

How strong is this variable as a predictor of severe illness in children?
- ◯ Strong
- ◯ Moderate
- ◯ Unlikely

Can this variable be reliably measured (i.e. inter- and intra-rater reliability)
- ◯ Highly reliable
- ◯ Moderately reliably
- ◯ Unreliably

How much training will be required to collect the variable?
- ◯ Extensive
- ◯ Moderate
- ◯ Minimal

How much will this variable overlap with other variables?
- ◯ Significant overlap
- ◯ Minimal overlap
- ◯ Not applicable

Which variables will this variable overlap with? ______________________________

How common is this variable abnormal?
- ◯ Frequently
- ◯ Less than half the time
- ◯ Rarely
- ◯ Not applicable

Airway Breathing

Apnea (observed, reported)

Should this variable be collected?
◯ Yes
◯ No

How strong is this variable as a predictor of severe illness in children?
◯ Strong
◯ Moderate
◯ Unlikely

Can this variable be reliably measured (i.e. inter- and intra-rater reliability)
◯ Highly reliable
◯ Moderately reliably
◯ Unreliably

How much training will be required to collect the variable?
◯ Extensive
◯ Moderate
◯ Minimal

How much will this variable overlap with other variables?
◯ Significant overlap
◯ Minimal overlap
◯ Not applicable

Which variables will this variable overlap with?
____

How common is this variable abnormal?
◯ Frequently
◯ Less than half the time
◯ Rarely
◯ Not applicable

Other comments.
____

Difficulty breathing (reported)

Should this variable be collected?
◯ Yes
◯ No

How strong is this variable as a predictor of severe illness in children?
◯ Strong
◯ Moderate
◯ Unlikely

Can this variable be reliably measured (i.e. inter- and intra-rater reliability)
◯ Highly reliable
◯ Moderately reliably
◯ Unreliably

How much training will be required to collect the variable?
◯ Extensive
◯ Moderate
◯ Minimal

How much will this variable overlap with other variables?
◯ Significant overlap
◯ Minimal overlap
◯ Not applicable

Which variables will this variable overlap with?
____

How common is this variable abnormal?
◯ Frequently
◯ Less than half the time
◯ Rarely
◯ Not applicable

Other comments.
____

Difficulty breathing (observed)

Should this variable be collected?
◯ Yes
◯ No

How strong is this variable as a predictor of severe illness in children?
◯ Strong
◯ Moderate
◯ Unlikely

Can this variable be reliably measured (i.e. inter- and intra-rater reliability)
◯ Highly reliable
◯ Moderately reliably
◯ Unreliably

How much training will be required to collect the variable?
◯ Extensive
◯ Moderate
◯ Minimal

How much will this variable overlap with other variables?
◯ Significant overlap
◯ Minimal overlap
◯ Not applicable

Which variables will this variable overlap with?
____

How common is this variable abnormal?
◯ Frequently
◯ Less than half the time
◯ Rarely
◯ Not applicable

Other comments.
____

Central Cyanosis

Should this variable be collected?
◯ Yes
◯ No

How strong is this variable as a predictor of severe illness in children?
◯ Strong
◯ Moderate
◯ Unlikely

Can this variable be reliably measured (i.e. inter- and intra-rater reliability)
◯ Highly reliable
◯ Moderately reliably
◯ Unreliably

How much training will be required to collect the variable?
◯ Extensive
◯ Moderate
◯ Minimal

How much will this variable overlap with other variables?
◯ Significant overlap
◯ Minimal overlap
◯ Not applicable

Which variables will this variable overlap with? __________________________________

How common is this variable abnormal?
◯ Frequently
◯ Less than half the time
◯ Rarely
◯ Not applicable

Other comments. ____

Chest in-drawing

Should this variable be collected?
◯ Yes
◯ No

How strong is this variable as a predictor of severe illness in children?
◯ Strong
◯ Moderate
◯ Unlikely

Can this variable be reliably measured (i.e. inter- and intra-rater reliability)
◯ Highly reliable
◯ Moderately reliably
◯ Unreliably

How much training will be required to collect the variable?
◯ Extensive
◯ Moderate
◯ Minimal

How much will this variable overlap with other variables?
◯ Significant overlap
◯ Minimal overlap
◯ Not applicable

Which variables will this variable overlap with? __________________________________

How common is this variable abnormal?
◯ Frequently
◯ Less than half the time
◯ Rarely
◯ Not applicable

Other comments. ____

Obstructed breathing

Should this variable be collected?
◯ Yes
◯ No

How strong is this variable as a predictor of severe illness in children?
◯ Strong
◯ Moderate
◯ Unlikely

Can this variable be reliably measured (i.e. inter- and intra-rater reliability)
◯ Highly reliable
◯ Moderately reliably
◯ Unreliably

How much training will be required to collect the variable?

◯ Extensive
◯ Moderate
◯ Minimal

How much will this variable overlap with other variables?

◯ Significant overlap
◯ Minimal overlap
◯ Not applicable

Which variables will this variable overlap with?

____

How common is this variable abnormal?

◯ Frequently
◯ Less than half the time
◯ Rarely
◯ Not applicable

Other comments.

____

Nasal flaring

Should this variable be collected?

◯ Yes
◯ No

How strong is this variable as a predictor of severe illness in children?

◯ Strong
◯ Moderate
◯ Unlikely

Can this variable be reliably measured (i.e. inter- and intra-rater reliability)

◯ Highly reliable
◯ Moderately reliably
◯ Unreliably

How much training will be required to collect the variable?

◯ Extensive
◯ Moderate
◯ Minimal

How much will this variable overlap with other variables?

◯ Significant overlap
◯ Minimal overlap
◯ Not applicable

Which variables will this variable overlap with?

____

How common is this variable abnormal?

◯ Frequently
◯ Less than half the time
◯ Rarely
◯ Not applicable

Other comments.

____

Grunting

Should this variable be collected?

- ◯ Yes
- ◯ No

How strong is this variable as a predictor of severe illness in children?

- ◯ Strong
- ◯ Moderate
- ◯ Unlikely

Can this variable be reliably measured (i.e. inter- and intra-rater reliability)

- ◯ Highly reliable
- ◯ Moderately reliably
- ◯ Unreliably

How much training will be required to collect the variable?

- ◯ Extensive
- ◯ Moderate
- ◯ Minimal

How much will this variable overlap with other variables?

- ◯ Significant overlap
- ◯ Minimal overlap
- ◯ Not applicable

Which variables will this variable overlap with? ______________________________

How common is this variable abnormal?

- ◯ Frequently
- ◯ Less than half the time
- ◯ Rarely
- ◯ Not applicable

Other comments. ______________________________________

Head bobbing/nodding

Should this variable be collected?

- ◯ Yes
- ◯ No

How strong is this variable as a predictor of severe illness in children?

- ◯ Strong
- ◯ Moderate
- ◯ Unlikely

Can this variable be reliably measured (i.e. inter- and intra-rater reliability)

- ◯ Highly reliable
- ◯ Moderately reliably
- ◯ Unreliably

How much training will be required to collect the variable?

- ◯ Extensive
- ◯ Moderate
- ◯ Minimal

How much will this variable overlap with other variables?

- ◯ Significant overlap
- ◯ Minimal overlap
- ◯ Not applicable

Which variables will this variable overlap with? ______________________________

How common is this variable abnormal?

- ◯ Frequently
- ◯ Less than half the time
- ◯ Rarely
- ◯ Not applicable

Other comments. ______________________

Crepitations

Should this variable be collected?
- ◯ Yes
- ◯ No

How strong is this variable as a predictor of severe illness in children?
- ◯ Strong
- ◯ Moderate
- ◯ Unlikely

Can this variable be reliably measured (i.e. inter- and intra-rater reliability)
- ◯ Highly reliable
- ◯ Moderately reliably
- ◯ Unreliably

How much training will be required to collect the variable?
- ◯ Extensive
- ◯ Moderate
- ◯ Minimal

How much will this variable overlap with other variables?
- ◯ Significant overlap
- ◯ Minimal overlap
- ◯ Not applicable

Which variables will this variable overlap with? ______________________

How common is this variable abnormal?
- ◯ Frequently
- ◯ Less than half the time
- ◯ Rarely
- ◯ Not applicable

Other comments. ______________________

Stridor

Should this variable be collected?
- ◯ Yes
- ◯ No

How strong is this variable as a predictor of severe illness in children?
- ◯ Strong
- ◯ Moderate
- ◯ Unlikely

Can this variable be reliably measured (i.e. inter- and intra-rater reliability)
- ◯ Highly reliable
- ◯ Moderately reliably
- ◯ Unreliably

How much training will be required to collect the variable?
- ◯ Extensive
- ◯ Moderate
- ◯ Minimal

How much will this variable overlap with other variables?
- ◯ Significant overlap
- ◯ Minimal overlap
- ◯ Not applicable

Which variables will this variable overlap with? ______________________

How common is this variable abnormal?

◯ Frequently
◯ Less than half the time
◯ Rarely
◯ Not applicable

Other comments.

____

Wheezing

Should this variable be collected?

◯ Yes
◯ No

How strong is this variable as a predictor of severe illness in children?

◯ Strong
◯ Moderate
◯ Unlikely

Can this variable be reliably measured (i.e. inter- and intra-rater reliability)

◯ Highly reliable
◯ Moderately reliably
◯ Unreliably

How much training will be required to collect the variable?

◯ Extensive
◯ Moderate
◯ Minimal

How much will this variable overlap with other variables?

◯ Significant overlap
◯ Minimal overlap
◯ Not applicable

Which variables will this variable overlap with?

____

How common is this variable abnormal?

◯ Frequently
◯ Less than half the time
◯ Rarely
◯ Not applicable

Other comments.

____

Respiratory effort

Should this variable be collected?

◯ Yes
◯ No

How strong is this variable as a predictor of severe illness in children?

◯ Strong
◯ Moderate
◯ Unlikely

Can this variable be reliably measured (i.e. inter- and intra-rater reliability)

◯ Highly reliable
◯ Moderately reliably
◯ Unreliably

How much training will be required to collect the variable?

◯ Extensive
◯ Moderate
◯ Minimal

How much will this variable overlap with other variables?
- ◯ Significant overlap
- ◯ Minimal overlap
- ◯ Not applicable

Which variables will this variable overlap with? ______________________________

How common is this variable abnormal?
- ◯ Frequently
- ◯ Less than half the time
- ◯ Rarely
- ◯ Not applicable

Other comments.

____

Fast breathing (reported)

Should this variable be collected?
- ◯ Yes
- ◯ No

How strong is this variable as a predictor of severe illness in children?
- ◯ Strong
- ◯ Moderate
- ◯ Unlikely

Can this variable be reliably measured (i.e. inter- and intra-rater reliability)
- ◯ Highly reliable
- ◯ Moderately reliably
- ◯ Unreliably

How much training will be required to collect the variable?
- ◯ Extensive
- ◯ Moderate
- ◯ Minimal

How much will this variable overlap with other variables?
- ◯ Significant overlap
- ◯ Minimal overlap
- ◯ Not applicable

Which variables will this variable overlap with? ______________________________

How common is this variable abnormal?
- ◯ Frequently
- ◯ Less than half the time
- ◯ Rarely
- ◯ Not applicable

Other comments.

____

Fast breathing (determined to be above >60BPM)

Should this variable be collected?
- ◯ Yes
- ◯ No

How strong is this variable as a predictor of severe illness in children?
- ◯ Strong
- ◯ Moderate
- ◯ Unlikely

Can this variable be reliably measured (i.e. inter- and intra-rater reliability)
- ◯ Highly reliable
- ◯ Moderately reliably
- ◯ Unreliably

How much training will be required to collect the variable?

- ◯ Extensive
- ◯ Moderate
- ◯ Minimal

How much will this variable overlap with other variables?

- ◯ Significant overlap
- ◯ Minimal overlap
- ◯ Not applicable

Which variables will this variable overlap with? __________________________________

How common is this variable abnormal?

- ◯ Frequently
- ◯ Less than half the time
- ◯ Rarely
- ◯ Not applicable

Other comments.

____

Circulation

Capillary refill time

Should this variable be collected?

- ◯ Yes
- ◯ No

How strong is this variable as a predictor of severe illness in children?

- ◯ Strong
- ◯ Moderate
- ◯ Unlikely

Can this variable be reliably measured (i.e. inter- and intra-rater reliability)

- ◯ Highly reliable
- ◯ Moderately reliably
- ◯ Unreliably

How much training will be required to collect the variable?

- ◯ Extensive
- ◯ Moderate
- ◯ Minimal

How much will this variable overlap with other variables?

- ◯ Significant overlap
- ◯ Minimal overlap
- ◯ Not applicable

Which variables will this variable overlap with? ________________________

How common is this variable abnormal?

- ◯ Frequently
- ◯ Less than half the time
- ◯ Rarely
- ◯ Not applicable

Other comments.

____

Skin cold (cool peripheries)

Should this variable be collected?

- ◯ Yes
- ◯ No

How strong is this variable as a predictor of severe illness in children?

- ◯ Strong
- ◯ Moderate
- ◯ Unlikely

Can this variable be reliably measured (i.e. inter- and intra-rater reliability)

- ◯ Highly reliable
- ◯ Moderately reliably
- ◯ Unreliably

How much training will be required to collect the variable?

- ◯ Extensive
- ◯ Moderate
- ◯ Minimal

How much will this variable overlap with other variables?

- ◯ Significant overlap
- ◯ Minimal overlap
- ◯ Not applicable

Which variables will this variable overlap with? ________________________

How common is this variable abnormal?

◯ Frequently
◯ Less than half the time
◯ Rarely
◯ Not applicable

Other comments.

____

Weak and fast pulse

Should this variable be collected?

◯ Yes
◯ No

How strong is this variable as a predictor of severe illness in children?

◯ Strong
◯ Moderate
◯ Unlikely

Can this variable be reliably measured (i.e. inter- and intra-rater reliability)

◯ Highly reliable
◯ Moderately reliably
◯ Unreliably

How much training will be required to collect the variable?

◯ Extensive
◯ Moderate
◯ Minimal

How much will this variable overlap with other variables?

◯ Significant overlap
◯ Minimal overlap
◯ Not applicable

Which variables will this variable overlap with?

____

How common is this variable abnormal?

◯ Frequently
◯ Less than half the time
◯ Rarely
◯ Not applicable

Other comments.

____

Pallor - Palmar, oral, conjunctival

Should this variable be collected?

◯ Yes
◯ No

How strong is this variable as a predictor of severe illness in children?

◯ Strong
◯ Moderate
◯ Unlikely

Can this variable be reliably measured (i.e. inter- and intra-rater reliability)

◯ Highly reliable
◯ Moderately reliably
◯ Unreliably

How much training will be required to collect the variable?

◯ Extensive
◯ Moderate
◯ Minimal

How much will this variable overlap with other variables?

- ◯ Significant overlap
- ◯ Minimal overlap
- ◯ Not applicable

Which variables will this variable overlap with? __________________________________

How common is this variable abnormal?

- ◯ Frequently
- ◯ Less than half the time
- ◯ Rarely
- ◯ Not applicable

Other comments. ____

Dehydration

Skin turgor - pinching of the skin of abdomen/hand

Should this variable be collected?
- ◯ Yes
- ◯ No

How strong is this variable as a predictor of severe illness in children?
- ◯ Strong
- ◯ Moderate
- ◯ Unlikely

Can this variable be reliably measured (i.e. inter- and intra-rater reliability)
- ◯ Highly reliable
- ◯ Moderately reliably
- ◯ Unreliably

How much training will be required to collect the variable?
- ◯ Extensive
- ◯ Moderate
- ◯ Minimal

How much will this variable overlap with other variables?
- ◯ Significant overlap
- ◯ Minimal overlap
- ◯ Not applicable

Which variables will this variable overlap with? ______________________________

How common is this variable abnormal?
- ◯ Frequently
- ◯ Less than half the time
- ◯ Rarely
- ◯ Not applicable

Other comments. _____________________________________

Sunken eyes

Should this variable be collected?
- ◯ Yes
- ◯ No

How strong is this variable as a predictor of severe illness in children?
- ◯ Strong
- ◯ Moderate
- ◯ Unlikely

Can this variable be reliably measured (i.e. inter- and intra-rater reliability)
- ◯ Highly reliable
- ◯ Moderately reliably
- ◯ Unreliably

How much training will be required to collect the variable?
- ◯ Extensive
- ◯ Moderate
- ◯ Minimal

How much will this variable overlap with other variables?
- ◯ Significant overlap
- ◯ Minimal overlap
- ◯ Not applicable

Which variables will this variable overlap with? ______________________________

How common is this variable abnormal?

- ◯ Frequently
- ◯ Less than half the time
- ◯ Rarely
- ◯ Not applicable

Other comments. ______________________________

Depressed fontanelle

Should this variable be collected?

- ◯ Yes
- ◯ No

How strong is this variable as a predictor of severe illness in children?

- ◯ Strong
- ◯ Moderate
- ◯ Unlikely

Can this variable be reliably measured (i.e. inter- and intra-rater reliability)

- ◯ Highly reliable
- ◯ Moderately reliably
- ◯ Unreliably

How much training will be required to collect the variable?

- ◯ Extensive
- ◯ Moderate
- ◯ Minimal

How much will this variable overlap with other variables?

- ◯ Significant overlap
- ◯ Minimal overlap
- ◯ Not applicable

Which variables will this variable overlap with? ______________________________

How common is this variable abnormal?

- ◯ Frequently
- ◯ Less than half the time
- ◯ Rarely
- ◯ Not applicable

Other comments. ______________________________

No tears when crying

Should this variable be collected?

- ◯ Yes
- ◯ No

How strong is this variable as a predictor of severe illness in children?

- ◯ Strong
- ◯ Moderate
- ◯ Unlikely

Can this variable be reliably measured (i.e. inter- and intra-rater reliability)

- ◯ Highly reliable
- ◯ Moderately reliably
- ◯ Unreliably

How much training will be required to collect the variable?

- ◯ Extensive
- ◯ Moderate
- ◯ Minimal

How much will this variable overlap with other variables?

- ◯ Significant overlap
- ◯ Minimal overlap
- ◯ Not applicable

Which variables will this variable overlap with? ______________________________

How common is this variable abnormal?

- ◯ Frequently
- ◯ Less than half the time
- ◯ Rarely
- ◯ Not applicable

Other comments.

____

Reduced urine production

Should this variable be collected?

- ◯ Yes
- ◯ No

How strong is this variable as a predictor of severe illness in children?

- ◯ Strong
- ◯ Moderate
- ◯ Unlikely

Can this variable be reliably measured (i.e. inter- and intra-rater reliability)

- ◯ Highly reliable
- ◯ Moderately reliably
- ◯ Unreliably

How much training will be required to collect the variable?

- ◯ Extensive
- ◯ Moderate
- ◯ Minimal

How much will this variable overlap with other variables?

- ◯ Significant overlap
- ◯ Minimal overlap
- ◯ Not applicable

Which variables will this variable overlap with? ______________________________

How common is this variable abnormal?

- ◯ Frequently
- ◯ Less than half the time
- ◯ Rarely
- ◯ Not applicable

Other comments.

____

Dry oral mucosa

Should this variable be collected?

- ◯ Yes
- ◯ No

How strong is this variable as a predictor of severe illness in children?

- ◯ Strong
- ◯ Moderate
- ◯ Unlikely

Can this variable be reliably measured (i.e. inter- and intra-rater reliability)

- ◯ Highly reliable
- ◯ Moderately reliably
- ◯ Unreliably

How much training will be required to collect the variable?
- ◯ Extensive
- ◯ Moderate
- ◯ Minimal

How much will this variable overlap with other variables?
- ◯ Significant overlap
- ◯ Minimal overlap
- ◯ Not applicable

Which variables will this variable overlap with? ______________________________

How common is this variable abnormal?
- ◯ Frequently
- ◯ Less than half the time
- ◯ Rarely
- ◯ Not applicable

Other comments.

____

Neurological

Irritability, restlessness

Should this variable be collected?
- ◯ Yes
- ◯ No

How strong is this variable as a predictor of severe illness in children?
- ◯ Strong
- ◯ Moderate
- ◯ Unlikely

Can this variable be reliably measured (i.e. inter- and intra-rater reliability)
- ◯ Highly reliable
- ◯ Moderately reliably
- ◯ Unreliably

How much training will be required to collect the variable?
- ◯ Extensive
- ◯ Moderate
- ◯ Minimal

How much will this variable overlap with other variables?
- ◯ Significant overlap
- ◯ Minimal overlap
- ◯ Not applicable

Which variables will this variable overlap with? ______________________

How common is this variable abnormal?
- ◯ Frequently
- ◯ Less than half the time
- ◯ Rarely
- ◯ Not applicable

Other comments.

____

Consolability

Should this variable be collected?
- ◯ Yes
- ◯ No

How strong is this variable as a predictor of severe illness in children?
- ◯ Strong
- ◯ Moderate
- ◯ Unlikely

Can this variable be reliably measured (i.e. inter- and intra-rater reliability)
- ◯ Highly reliable
- ◯ Moderately reliably
- ◯ Unreliably

How much training will be required to collect the variable?
- ◯ Extensive
- ◯ Moderate
- ◯ Minimal

How much will this variable overlap with other variables?
- ◯ Significant overlap
- ◯ Minimal overlap
- ◯ Not applicable

Which variables will this variable overlap with? ______________________

How common is this variable abnormal?

◯ Frequently
◯ Less than half the time
◯ Rarely
◯ Not applicable

Other comments.

____

Sleepiness/drowsiness/unconscious

Should this variable be collected?

◯ Yes
◯ No

How strong is this variable as a predictor of severe illness in children?

◯ Strong
◯ Moderate
◯ Unlikely

Can this variable be reliably measured (i.e. inter- and intra-rater reliability)

◯ Highly reliable
◯ Moderately reliably
◯ Unreliably

How much training will be required to collect the variable?

◯ Extensive
◯ Moderate
◯ Minimal

How much will this variable overlap with other variables?

◯ Significant overlap
◯ Minimal overlap
◯ Not applicable

Which variables will this variable overlap with?

____

How common is this variable abnormal?

◯ Frequently
◯ Less than half the time
◯ Rarely
◯ Not applicable

Other comments.

____

Ease of wakening

Should this variable be collected?

◯ Yes
◯ No

How strong is this variable as a predictor of severe illness in children?

◯ Strong
◯ Moderate
◯ Unlikely

Can this variable be reliably measured (i.e. inter- and intra-rater reliability)

◯ Highly reliable
◯ Moderately reliably
◯ Unreliably

How much training will be required to collect the variable?

◯ Extensive
◯ Moderate
◯ Minimal

How much will this variable overlap with other variables?
- ◯ Significant overlap
- ◯ Minimal overlap
- ◯ Not applicable

Which variables will this variable overlap with? ________________________________

How common is this variable abnormal?
- ◯ Frequently
- ◯ Less than half the time
- ◯ Rarely
- ◯ Not applicable

Other comments. ________________________________

Level of consciousness

Should this variable be collected?
- ◯ Yes
- ◯ No

How strong is this variable as a predictor of severe illness in children?
- ◯ Strong
- ◯ Moderate
- ◯ Unlikely

How do you think we should assess LOC?
- ◯ AVPU
- ◯ Blantyre Coma Scale
- ◯ Glasgow Coma Scale
- ◯ Ballard Score

Can this variable be reliably measured (i.e. inter- and intra-rater reliability)
- ◯ Highly reliable
- ◯ Moderately reliably
- ◯ Unreliably

How much training will be required to collect the variable?
- ◯ Extensive
- ◯ Moderate
- ◯ Minimal

How much will this variable overlap with other variables?
- ◯ Significant overlap
- ◯ Minimal overlap
- ◯ Not applicable

Which variables will this variable overlap with? ________________________________

How common is this variable abnormal?
- ◯ Frequently
- ◯ Less than half the time
- ◯ Rarely
- ◯ Not applicable

Other comments. ________________________________

Lethargy

Should this variable be collected?
- ◯ Yes
- ◯ No

How strong is this variable as a predictor of severe illness in children?
- ◯ Strong
- ◯ Moderate
- ◯ Unlikely

Can this variable be reliably measured (i.e. inter- and intra-rater reliability)
- ◯ Highly reliable
- ◯ Moderately reliably
- ◯ Unreliably

How much training will be required to collect the variable?
- ◯ Extensive
- ◯ Moderate
- ◯ Minimal

How much will this variable overlap with other variables?
- ◯ Significant overlap
- ◯ Minimal overlap
- ◯ Not applicable

Which variables will this variable overlap with? ______________________________

How common is this variable abnormal?
- ◯ Frequently
- ◯ Less than half the time
- ◯ Rarely
- ◯ Not applicable

Other comments.

____

Convulsions (reported, history)

Should this variable be collected?
- ◯ Yes
- ◯ No

How strong is this variable as a predictor of severe illness in children?
- ◯ Strong
- ◯ Moderate
- ◯ Unlikely

Can this variable be reliably measured (i.e. inter- and intra-rater reliability)
- ◯ Highly reliable
- ◯ Moderately reliably
- ◯ Unreliably

How much training will be required to collect the variable?
- ◯ Extensive
- ◯ Moderate
- ◯ Minimal

How much will this variable overlap with other variables?
- ◯ Significant overlap
- ◯ Minimal overlap
- ◯ Not applicable

Which variables will this variable overlap with? ______________________________

How common is this variable abnormal?
- ◯ Frequently
- ◯ Less than half the time
- ◯ Rarely
- ◯ Not applicable

Other comments. ____

Convulsing now, actively

Should this variable be collected?
- ◯ Yes
- ◯ No

How strong is this variable as a predictor of severe illness in children?
- ◯ Strong
- ◯ Moderate
- ◯ Unlikely

Can this variable be reliably measured (i.e. inter- and intra-rater reliability)
- ◯ Highly reliable
- ◯ Moderately reliably
- ◯ Unreliably

How much training will be required to collect the variable?
- ◯ Extensive
- ◯ Moderate
- ◯ Minimal

How much will this variable overlap with other variables?
- ◯ Significant overlap
- ◯ Minimal overlap
- ◯ Not applicable

Which variables will this variable overlap with? __________________________________

How common is this variable abnormal?
- ◯ Frequently
- ◯ Less than half the time
- ◯ Rarely
- ◯ Not applicable

Other comments. ____

Confusion

Should this variable be collected?
- ◯ Yes
- ◯ No

How strong is this variable as a predictor of severe illness in children?
- ◯ Strong
- ◯ Moderate
- ◯ Unlikely

Can this variable be reliably measured (i.e. inter- and intra-rater reliability)
- ◯ Highly reliable
- ◯ Moderately reliably
- ◯ Unreliably

How much training will be required to collect the variable?
- ◯ Extensive
- ◯ Moderate
- ◯ Minimal

How much will this variable overlap with other variables?
- ◯ Significant overlap
- ◯ Minimal overlap
- ◯ Not applicable

Which variables will this variable overlap with? __________________________________

How common is this variable abnormal?

◯ Frequently
◯ Less than half the time
◯ Rarely
◯ Not applicable

Other comments.

____

Stiff limbs

Should this variable be collected?

◯ Yes
◯ No

How strong is this variable as a predictor of severe illness in children?

◯ Strong
◯ Moderate
◯ Unlikely

Can this variable be reliably measured (i.e. inter- and intra-rater reliability)

◯ Highly reliable
◯ Moderately reliably
◯ Unreliably

How much training will be required to collect the variable?

◯ Extensive
◯ Moderate
◯ Minimal

How much will this variable overlap with other variables?

◯ Significant overlap
◯ Minimal overlap
◯ Not applicable

Which variables will this variable overlap with?

____

How common is this variable abnormal?

◯ Frequently
◯ Less than half the time
◯ Rarely
◯ Not applicable

Other comments.

____

Hypotonia

Should this variable be collected?

◯ Yes
◯ No

How strong is this variable as a predictor of severe illness in children?

◯ Strong
◯ Moderate
◯ Unlikely

Can this variable be reliably measured (i.e. inter- and intra-rater reliability)

◯ Highly reliable
◯ Moderately reliably
◯ Unreliably

How much training will be required to collect the variable?

◯ Extensive
◯ Moderate
◯ Minimal

How much will this variable overlap with other variables?

- ◯ Significant overlap
- ◯ Minimal overlap
- ◯ Not applicable

Which variables will this variable overlap with? ________________________________

How common is this variable abnormal?

- ◯ Frequently
- ◯ Less than half the time
- ◯ Rarely
- ◯ Not applicable

Other comments.

____

Mobility

Should this variable be collected?

- ◯ Yes
- ◯ No

How strong is this variable as a predictor of severe illness in children?

- ◯ Strong
- ◯ Moderate
- ◯ Unlikely

Can this variable be reliably measured (i.e. inter- and intra-rater reliability)

- ◯ Highly reliable
- ◯ Moderately reliably
- ◯ Unreliably

How much training will be required to collect the variable?

- ◯ Extensive
- ◯ Moderate
- ◯ Minimal

How much will this variable overlap with other variables?

- ◯ Significant overlap
- ◯ Minimal overlap
- ◯ Not applicable

Which variables will this variable overlap with? ________________________________

How common is this variable abnormal?

- ◯ Frequently
- ◯ Less than half the time
- ◯ Rarely
- ◯ Not applicable

Other comments.

____

Spontaneous movements/to stimulus

Should this variable be collected?

- ◯ Yes
- ◯ No

How strong is this variable as a predictor of severe illness in children?

- ◯ Strong
- ◯ Moderate
- ◯ Unlikely

Can this variable be reliably measured (i.e. inter- and intra-rater reliability)

- ◯ Highly reliable
- ◯ Moderately reliably
- ◯ Unreliably

How much training will be required to collect the variable?
- ◯ Extensive
- ◯ Moderate
- ◯ Minimal

How much will this variable overlap with other variables?
- ◯ Significant overlap
- ◯ Minimal overlap
- ◯ Not applicable

Which variables will this variable overlap with? ______________________

How common is this variable abnormal?
- ◯ Frequently
- ◯ Less than half the time
- ◯ Rarely
- ◯ Not applicable

Other comments. ______________________

Neck pain/stiffness

Should this variable be collected?
- ◯ Yes
- ◯ No

How strong is this variable as a predictor of severe illness in children?
- ◯ Strong
- ◯ Moderate
- ◯ Unlikely

Can this variable be reliably measured (i.e. inter- and intra-rater reliability)
- ◯ Highly reliable
- ◯ Moderately reliably
- ◯ Unreliably

How much training will be required to collect the variable?
- ◯ Extensive
- ◯ Moderate
- ◯ Minimal

How much will this variable overlap with other variables?
- ◯ Significant overlap
- ◯ Minimal overlap
- ◯ Not applicable

Which variables will this variable overlap with? ______________________

How common is this variable abnormal?
- ◯ Frequently
- ◯ Less than half the time
- ◯ Rarely
- ◯ Not applicable

Other comments. ______________________

Bulging fontanelles

Should this variable be collected?
- ◯ Yes
- ◯ No

How strong is this variable as a predictor of severe illness in children?
- ◯ Strong
- ◯ Moderate
- ◯ Unlikely

Can this variable be reliably measured (i.e. inter- and intra-rater reliability)
- ◯ Highly reliable
- ◯ Moderately reliably
- ◯ Unreliably

How much training will be required to collect the variable?
- ◯ Extensive
- ◯ Moderate
- ◯ Minimal

How much will this variable overlap with other variables?
- ◯ Significant overlap
- ◯ Minimal overlap
- ◯ Not applicable

Which variables will this variable overlap with? ______________________________

How common is this variable abnormal?
- ◯ Frequently
- ◯ Less than half the time
- ◯ Rarely
- ◯ Not applicable

Other comments. ______________________________

Focal neurology acute

Should this variable be collected?
- ◯ Yes
- ◯ No

How strong is this variable as a predictor of severe illness in children?
- ◯ Strong
- ◯ Moderate
- ◯ Unlikely

Can this variable be reliably measured (i.e. inter- and intra-rater reliability)
- ◯ Highly reliable
- ◯ Moderately reliably
- ◯ Unreliably

How much training will be required to collect the variable?
- ◯ Extensive
- ◯ Moderate
- ◯ Minimal

How much will this variable overlap with other variables?
- ◯ Significant overlap
- ◯ Minimal overlap
- ◯ Not applicable

Which variables will this variable overlap with? ______________________________

How common is this variable abnormal?
- ◯ Frequently
- ◯ Less than half the time
- ◯ Rarely
- ◯ Not applicable

Other comments.

____

Not able to drink or feed anything

Should this variable be collected?
- ◯ Yes
- ◯ No

How strong is this variable as a predictor of severe illness in children?
- ◯ Strong
- ◯ Moderate
- ◯ Unlikely

Can this variable be reliably measured (i.e. inter- and intra-rater reliability)
- ◯ Highly reliable
- ◯ Moderately reliably
- ◯ Unreliably

How much training will be required to collect the variable?
- ◯ Extensive
- ◯ Moderate
- ◯ Minimal

How much will this variable overlap with other variables?
- ◯ Significant overlap
- ◯ Minimal overlap
- ◯ Not applicable

Which variables will this variable overlap with? __________________________

How common is this variable abnormal?
- ◯ Frequently
- ◯ Less than half the time
- ◯ Rarely
- ◯ Not applicable

Other comments.

____

Not feeding well

Should this variable be collected?
- ◯ Yes
- ◯ No

How strong is this variable as a predictor of severe illness in children?
- ◯ Strong
- ◯ Moderate
- ◯ Unlikely

Can this variable be reliably measured (i.e. inter- and intra-rater reliability)
- ◯ Highly reliable
- ◯ Moderately reliably
- ◯ Unreliably

How much training will be required to collect the variable?
- ◯ Extensive
- ◯ Moderate
- ◯ Minimal

How much will this variable overlap with other variables?
- ◯ Significant overlap
- ◯ Minimal overlap
- ◯ Not applicable

Which variables will this variable overlap with? __________________________

How common is this variable abnormal?

◯ Frequently
◯ Less than half the time
◯ Rarely
◯ Not applicable

Other comments.

____

Not suckling/breastfeeding

Should this variable be collected?

◯ Yes
◯ No

How strong is this variable as a predictor of severe illness in children?

◯ Strong
◯ Moderate
◯ Unlikely

Can this variable be reliably measured (i.e. inter- and intra-rater reliability)

◯ Highly reliable
◯ Moderately reliably
◯ Unreliably

How much training will be required to collect the variable?

◯ Extensive
◯ Moderate
◯ Minimal

How much will this variable overlap with other variables?

◯ Significant overlap
◯ Minimal overlap
◯ Not applicable

Which variables will this variable overlap with?

____

How common is this variable abnormal?

◯ Frequently
◯ Less than half the time
◯ Rarely
◯ Not applicable

Other comments.

____

Infection

Fever

Should this variable be collected?

- ◯ Yes
- ◯ No

How strong is this variable as a predictor of severe illness in children?

- ◯ Strong
- ◯ Moderate
- ◯ Unlikely

Can this variable be reliably measured (i.e. inter- and intra-rater reliability)

- ◯ Highly reliable
- ◯ Moderately reliably
- ◯ Unreliably

How much training will be required to collect the variable?

- ◯ Extensive
- ◯ Moderate
- ◯ Minimal

How much will this variable overlap with other variables?

- ◯ Significant overlap
- ◯ Minimal overlap
- ◯ Not applicable

Which variables will this variable overlap with? __________________________

How common is this variable abnormal?

- ◯ Frequently
- ◯ Less than half the time
- ◯ Rarely
- ◯ Not applicable

Other comments.

____

Cough

Should this variable be collected?

- ◯ Yes
- ◯ No

How strong is this variable as a predictor of severe illness in children?

- ◯ Strong
- ◯ Moderate
- ◯ Unlikely

Can this variable be reliably measured (i.e. inter- and intra-rater reliability)

- ◯ Highly reliable
- ◯ Moderately reliably
- ◯ Unreliably

How much training will be required to collect the variable?

- ◯ Extensive
- ◯ Moderate
- ◯ Minimal

How much will this variable overlap with other variables?

- ◯ Significant overlap
- ◯ Minimal overlap
- ◯ Not applicable

Which variables will this variable overlap with? __________________________

How common is this variable abnormal?

- ◯ Frequently
- ◯ Less than half the time
- ◯ Rarely
- ◯ Not applicable

Other comments.

____

Runny nose

Should this variable be collected?

- ◯ Yes
- ◯ No

How strong is this variable as a predictor of severe illness in children?

- ◯ Strong
- ◯ Moderate
- ◯ Unlikely

Can this variable be reliably measured (i.e. inter- and intra-rater reliability)

- ◯ Highly reliable
- ◯ Moderately reliably
- ◯ Unreliably

How much training will be required to collect the variable?

- ◯ Extensive
- ◯ Moderate
- ◯ Minimal

How much will this variable overlap with other variables?

- ◯ Significant overlap
- ◯ Minimal overlap
- ◯ Not applicable

Which variables will this variable overlap with? ___________________________

How common is this variable abnormal?

- ◯ Frequently
- ◯ Less than half the time
- ◯ Rarely
- ◯ Not applicable

Other comments.

____

Rash

Should this variable be collected?

- ◯ Yes
- ◯ No

How strong is this variable as a predictor of severe illness in children?

- ◯ Strong
- ◯ Moderate
- ◯ Unlikely

Can this variable be reliably measured (i.e. inter- and intra-rater reliability)

- ◯ Highly reliable
- ◯ Moderately reliably
- ◯ Unreliably

How much training will be required to collect the variable?

- ◯ Extensive
- ◯ Moderate
- ◯ Minimal

How much will this variable overlap with other variables?
◯ Significant overlap
◯ Minimal overlap
◯ Not applicable

Which variables will this variable overlap with? ______________________

How common is this variable abnormal?
◯ Frequently
◯ Less than half the time
◯ Rarely
◯ Not applicable

Other comments. ______________________

Ear pain

Should this variable be collected?
◯ Yes
◯ No

How strong is this variable as a predictor of severe illness in children?
◯ Strong
◯ Moderate
◯ Unlikely

Can this variable be reliably measured (i.e. inter- and intra-rater reliability)
◯ Highly reliable
◯ Moderately reliably
◯ Unreliably

How much training will be required to collect the variable?
◯ Extensive
◯ Moderate
◯ Minimal

How much will this variable overlap with other variables?
◯ Significant overlap
◯ Minimal overlap
◯ Not applicable

Which variables will this variable overlap with? ______________________

How common is this variable abnormal?
◯ Frequently
◯ Less than half the time
◯ Rarely
◯ Not applicable

Other comments. ______________________

Ear discharge

Should this variable be collected?
◯ Yes
◯ No

How strong is this variable as a predictor of severe illness in children?
◯ Strong
◯ Moderate
◯ Unlikely

Can this variable be reliably measured (i.e. inter- and intra-rater reliability)
◯ Highly reliable
◯ Moderately reliably
◯ Unreliably

How much training will be required to collect the variable?
- ◯ Extensive
- ◯ Moderate
- ◯ Minimal

How much will this variable overlap with other variables?
- ◯ Significant overlap
- ◯ Minimal overlap
- ◯ Not applicable

Which variables will this variable overlap with? __________________________

How common is this variable abnormal?
- ◯ Frequently
- ◯ Less than half the time
- ◯ Rarely
- ◯ Not applicable

Other comments.

____

Tender swelling behind the ear

Should this variable be collected?
- ◯ Yes
- ◯ No

How strong is this variable as a predictor of severe illness in children?
- ◯ Strong
- ◯ Moderate
- ◯ Unlikely

Can this variable be reliably measured (i.e. inter- and intra-rater reliability)
- ◯ Highly reliable
- ◯ Moderately reliably
- ◯ Unreliably

How much training will be required to collect the variable?
- ◯ Extensive
- ◯ Moderate
- ◯ Minimal

How much will this variable overlap with other variables?
- ◯ Significant overlap
- ◯ Minimal overlap
- ◯ Not applicable

Which variables will this variable overlap with? __________________________

How common is this variable abnormal?
- ◯ Frequently
- ◯ Less than half the time
- ◯ Rarely
- ◯ Not applicable

Other comments.

____

Purulent drainage eyes

Should this variable be collected?
- ◯ Yes
- ◯ No

How strong is this variable as a predictor of severe illness in children?
- ◯ Strong
- ◯ Moderate
- ◯ Unlikely

Can this variable be reliably measured (i.e. inter- and intra-rater reliability)
- ◯ Highly reliable
- ◯ Moderately reliably
- ◯ Unreliably

How much training will be required to collect the variable?
- ◯ Extensive
- ◯ Moderate
- ◯ Minimal

How much will this variable overlap with other variables?
- ◯ Significant overlap
- ◯ Minimal overlap
- ◯ Not applicable

Which variables will this variable overlap with? ______________________________

How common is this variable abnormal?
- ◯ Frequently
- ◯ Less than half the time
- ◯ Rarely
- ◯ Not applicable

Other comments.

____

Conjunctivitis

Should this variable be collected?
- ◯ Yes
- ◯ No

How strong is this variable as a predictor of severe illness in children?
- ◯ Strong
- ◯ Moderate
- ◯ Unlikely

Can this variable be reliably measured (i.e. inter- and intra-rater reliability)
- ◯ Highly reliable
- ◯ Moderately reliably
- ◯ Unreliably

How much training will be required to collect the variable?
- ◯ Extensive
- ◯ Moderate
- ◯ Minimal

How much will this variable overlap with other variables?
- ◯ Significant overlap
- ◯ Minimal overlap
- ◯ Not applicable

Which variables will this variable overlap with? ______________________________

How common is this variable abnormal?
- ◯ Frequently
- ◯ Less than half the time
- ◯ Rarely
- ◯ Not applicable

Other comments.

____

Skin pustules

Should this variable be collected?
- ◯ Yes
- ◯ No

How strong is this variable as a predictor of severe illness in children?
- ◯ Strong
- ◯ Moderate
- ◯ Unlikely

Can this variable be reliably measured (i.e. inter- and intra-rater reliability)
- ◯ Highly reliable
- ◯ Moderately reliably
- ◯ Unreliably

How much training will be required to collect the variable?
- ◯ Extensive
- ◯ Moderate
- ◯ Minimal

How much will this variable overlap with other variables?
- ◯ Significant overlap
- ◯ Minimal overlap
- ◯ Not applicable

Which variables will this variable overlap with? ______________________________

How common is this variable abnormal?
- ◯ Frequently
- ◯ Less than half the time
- ◯ Rarely
- ◯ Not applicable

Other comments.

____

Umbilicus is red

Should this variable be collected?
- ◯ Yes
- ◯ No

How strong is this variable as a predictor of severe illness in children?
- ◯ Strong
- ◯ Moderate
- ◯ Unlikely

Can this variable be reliably measured (i.e. inter- and intra-rater reliability)
- ◯ Highly reliable
- ◯ Moderately reliably
- ◯ Unreliably

How much training will be required to collect the variable?
- ◯ Extensive
- ◯ Moderate
- ◯ Minimal

How much will this variable overlap with other variables?
- ◯ Significant overlap
- ◯ Minimal overlap
- ◯ Not applicable

Which variables will this variable overlap with? ______________________________

How common is this variable abnormal?

- ◯ Frequently
- ◯ Less than half the time
- ◯ Rarely
- ◯ Not applicable

Other comments.

____

Umbilicus is draining

Should this variable be collected?

- ◯ Yes
- ◯ No

How strong is this variable as a predictor of severe illness in children?

- ◯ Strong
- ◯ Moderate
- ◯ Unlikely

Can this variable be reliably measured (i.e. inter- and intra-rater reliability)

- ◯ Highly reliable
- ◯ Moderately reliably
- ◯ Unreliably

How much training will be required to collect the variable?

- ◯ Extensive
- ◯ Moderate
- ◯ Minimal

How much will this variable overlap with other variables?

- ◯ Significant overlap
- ◯ Minimal overlap
- ◯ Not applicable

Which variables will this variable overlap with? ______________________________

How common is this variable abnormal?

- ◯ Frequently
- ◯ Less than half the time
- ◯ Rarely
- ◯ Not applicable

Other comments.

____

Gastrointestinal Urinary

Diarrhea

Should this variable be collected?
- ◯ Yes
- ◯ No

How strong is this variable as a predictor of severe illness in children?
- ◯ Strong
- ◯ Moderate
- ◯ Unlikely

Can this variable be reliably measured (i.e. inter- and intra-rater reliability)
- ◯ Highly reliable
- ◯ Moderately reliably
- ◯ Unreliably

How much training will be required to collect the variable?
- ◯ Extensive
- ◯ Moderate
- ◯ Minimal

How much will this variable overlap with other variables?
- ◯ Significant overlap
- ◯ Minimal overlap
- ◯ Not applicable

Which variables will this variable overlap with? ______________________________

How common is this variable abnormal?
- ◯ Frequently
- ◯ Less than half the time
- ◯ Rarely
- ◯ Not applicable

Other comments. ______________________________________

Blood in stool (dysentery)

Should this variable be collected?
- ◯ Yes
- ◯ No

How strong is this variable as a predictor of severe illness in children?
- ◯ Strong
- ◯ Moderate
- ◯ Unlikely

Can this variable be reliably measured (i.e. inter- and intra-rater reliability)
- ◯ Highly reliable
- ◯ Moderately reliably
- ◯ Unreliably

How much training will be required to collect the variable?
- ◯ Extensive
- ◯ Moderate
- ◯ Minimal

How much will this variable overlap with other variables?
- ◯ Significant overlap
- ◯ Minimal overlap
- ◯ Not applicable

Which variables will this variable overlap with? ______________________________

How common is this variable abnormal?

◯ Frequently
◯ Less than half the time
◯ Rarely
◯ Not applicable

Other comments.

____

Vomiting

Should this variable be collected?

◯ Yes
◯ No

How strong is this variable as a predictor of severe illness in children?

◯ Strong
◯ Moderate
◯ Unlikely

Can this variable be reliably measured (i.e. inter- and intra-rater reliability)

◯ Highly reliable
◯ Moderately reliably
◯ Unreliably

How much training will be required to collect the variable?

◯ Extensive
◯ Moderate
◯ Minimal

How much will this variable overlap with other variables?

◯ Significant overlap
◯ Minimal overlap
◯ Not applicable

Which variables will this variable overlap with?

____

How common is this variable abnormal?

◯ Frequently
◯ Less than half the time
◯ Rarely
◯ Not applicable

Other comments.

____

Abdominal pain

Should this variable be collected?

◯ Yes
◯ No

How strong is this variable as a predictor of severe illness in children?

◯ Strong
◯ Moderate
◯ Unlikely

Can this variable be reliably measured (i.e. inter- and intra-rater reliability)

◯ Highly reliable
◯ Moderately reliably
◯ Unreliably

How much training will be required to collect the variable?

◯ Extensive
◯ Moderate
◯ Minimal

How much will this variable overlap with other variables?
◯ Significant overlap
◯ Minimal overlap
◯ Not applicable

Which variables will this variable overlap with? __________________________________

How common is this variable abnormal?
◯ Frequently
◯ Less than half the time
◯ Rarely
◯ Not applicable

Other comments. ____

Foul-smelling urine

Should this variable be collected?
◯ Yes
◯ No

How strong is this variable as a predictor of severe illness in children?
◯ Strong
◯ Moderate
◯ Unlikely

Can this variable be reliably measured (i.e. inter- and intra-rater reliability)
◯ Highly reliable
◯ Moderately reliably
◯ Unreliably

How much training will be required to collect the variable?
◯ Extensive
◯ Moderate
◯ Minimal

How much will this variable overlap with other variables?
◯ Significant overlap
◯ Minimal overlap
◯ Not applicable

Which variables will this variable overlap with? __________________________________

How common is this variable abnormal?
◯ Frequently
◯ Less than half the time
◯ Rarely
◯ Not applicable

Other comments. ____

Malnutrition

Swelling of both feet (peripheral edema)

Should this variable be collected?
- ◯ Yes
- ◯ No

How strong is this variable as a predictor of severe illness in children?
- ◯ Strong
- ◯ Moderate
- ◯ Unlikely

Can this variable be reliably measured (i.e. inter- and intra-rater reliability)
- ◯ Highly reliable
- ◯ Moderately reliably
- ◯ Unreliably

How much training will be required to collect the variable?
- ◯ Extensive
- ◯ Moderate
- ◯ Minimal

How much will this variable overlap with other variables?
- ◯ Significant overlap
- ◯ Minimal overlap
- ◯ Not applicable

Which variables will this variable overlap with? ______________________________

How common is this variable abnormal?
- ◯ Frequently
- ◯ Less than half the time
- ◯ Rarely
- ◯ Not applicable

Other comments. ______________________________________

Visible severe wasting (marasmus)

Should this variable be collected?
- ◯ Yes
- ◯ No

How strong is this variable as a predictor of severe illness in children?
- ◯ Strong
- ◯ Moderate
- ◯ Unlikely

Can this variable be reliably measured (i.e. inter- and intra-rater reliability)
- ◯ Highly reliable
- ◯ Moderately reliably
- ◯ Unreliably

How much training will be required to collect the variable?
- ◯ Extensive
- ◯ Moderate
- ◯ Minimal

How much will this variable overlap with other variables?
- ◯ Significant overlap
- ◯ Minimal overlap
- ◯ Not applicable

Which variables will this variable overlap with? ______________________________

How common is this variable abnormal?

- ◯ Frequently
- ◯ Less than half the time
- ◯ Rarely
- ◯ Not applicable

Other comments.

____

Abdominal distension

Should this variable be collected?

- ◯ Yes
- ◯ No

How strong is this variable as a predictor of severe illness in children?

- ◯ Strong
- ◯ Moderate
- ◯ Unlikely

Can this variable be reliably measured (i.e. inter- and intra-rater reliability)

- ◯ Highly reliable
- ◯ Moderately reliably
- ◯ Unreliably

How much training will be required to collect the variable?

- ◯ Extensive
- ◯ Moderate
- ◯ Minimal

How much will this variable overlap with other variables?

- ◯ Significant overlap
- ◯ Minimal overlap
- ◯ Not applicable

Which variables will this variable overlap with? __________________________

How common is this variable abnormal?

- ◯ Frequently
- ◯ Less than half the time
- ◯ Rarely
- ◯ Not applicable

Other comments.

____

Ulcers or white patches in the mouth (oral thrush)

Should this variable be collected?

- ◯ Yes
- ◯ No

How strong is this variable as a predictor of severe illness in children?

- ◯ Strong
- ◯ Moderate
- ◯ Unlikely

Can this variable be reliably measured (i.e. inter- and intra-rater reliability)

- ◯ Highly reliable
- ◯ Moderately reliably
- ◯ Unreliably

How much training will be required to collect the variable?

- ◯ Extensive
- ◯ Moderate
- ◯ Minimal

How much will this variable overlap with other variables?	◯ Significant overlap<br>◯ Minimal overlap<br>◯ Not applicable
Which variables will this variable overlap with?	__________________________________
How common is this variable abnormal?	◯ Frequently<br>◯ Less than half the time<br>◯ Rarely<br>◯ Not applicable
Other comments.	____

Trauma

Trauma or other surgical condition

Should this variable be collected?
- ◯ Yes
- ◯ No

How strong is this variable as a predictor of severe illness in children?
- ◯ Strong
- ◯ Moderate
- ◯ Unlikely

Can this variable be reliably measured (i.e. inter- and intra-rater reliability)
- ◯ Highly reliable
- ◯ Moderately reliably
- ◯ Unreliably

How much training will be required to collect the variable?
- ◯ Extensive
- ◯ Moderate
- ◯ Minimal

How much will this variable overlap with other variables?
- ◯ Significant overlap
- ◯ Minimal overlap
- ◯ Not applicable

Which variables will this variable overlap with? ______________________

How common is this variable abnormal?
- ◯ Frequently
- ◯ Less than half the time
- ◯ Rarely
- ◯ Not applicable

Other comments.

____

Eye injury

Should this variable be collected?
- ◯ Yes
- ◯ No

How strong is this variable as a predictor of severe illness in children?
- ◯ Strong
- ◯ Moderate
- ◯ Unlikely

Can this variable be reliably measured (i.e. inter- and intra-rater reliability)
- ◯ Highly reliable
- ◯ Moderately reliably
- ◯ Unreliably

How much training will be required to collect the variable?
- ◯ Extensive
- ◯ Moderate
- ◯ Minimal

How much will this variable overlap with other variables?
- ◯ Significant overlap
- ◯ Minimal overlap
- ◯ Not applicable

Which variables will this variable overlap with? ______________________

How common is this variable abnormal?
- ◯ Frequently
- ◯ Less than half the time
- ◯ Rarely
- ◯ Not applicable

Other comments.

____

Fracture

Should this variable be collected?
- ◯ Yes
- ◯ No

How strong is this variable as a predictor of severe illness in children?
- ◯ Strong
- ◯ Moderate
- ◯ Unlikely

Can this variable be reliably measured (i.e. inter- and intra-rater reliability)
- ◯ Highly reliable
- ◯ Moderately reliably
- ◯ Unreliably

How much training will be required to collect the variable?
- ◯ Extensive
- ◯ Moderate
- ◯ Minimal

How much will this variable overlap with other variables?
- ◯ Significant overlap
- ◯ Minimal overlap
- ◯ Not applicable

Which variables will this variable overlap with? ______________________

How common is this variable abnormal?
- ◯ Frequently
- ◯ Less than half the time
- ◯ Rarely
- ◯ Not applicable

Other comments.

____

Dislocation

Should this variable be collected?
- ◯ Yes
- ◯ No

How strong is this variable as a predictor of severe illness in children?
- ◯ Strong
- ◯ Moderate
- ◯ Unlikely

Can this variable be reliably measured (i.e. inter- and intra-rater reliability)
- ◯ Highly reliable
- ◯ Moderately reliably
- ◯ Unreliably

How much training will be required to collect the variable?
- ◯ Extensive
- ◯ Moderate
- ◯ Minimal

How much will this variable overlap with other variables?

- ◯ Significant overlap
- ◯ Minimal overlap
- ◯ Not applicable

Which variables will this variable overlap with? ______________________

How common is this variable abnormal?

- ◯ Frequently
- ◯ Less than half the time
- ◯ Rarely
- ◯ Not applicable

Other comments.

____

Hemorrhage

Should this variable be collected?

- ◯ Yes
- ◯ No

How strong is this variable as a predictor of severe illness in children?

- ◯ Strong
- ◯ Moderate
- ◯ Unlikely

Can this variable be reliably measured (i.e. inter- and intra-rater reliability)

- ◯ Highly reliable
- ◯ Moderately reliably
- ◯ Unreliably

How much training will be required to collect the variable?

- ◯ Extensive
- ◯ Moderate
- ◯ Minimal

How much will this variable overlap with other variables?

- ◯ Significant overlap
- ◯ Minimal overlap
- ◯ Not applicable

Which variables will this variable overlap with? ______________________

How common is this variable abnormal?

- ◯ Frequently
- ◯ Less than half the time
- ◯ Rarely
- ◯ Not applicable

Other comments.

____

Severe pain

Should this variable be collected?

- ◯ Yes
- ◯ No

How strong is this variable as a predictor of severe illness in children?

- ◯ Strong
- ◯ Moderate
- ◯ Unlikely

Can this variable be reliably measured (i.e. inter- and intra-rater reliability)

- ◯ Highly reliable
- ◯ Moderately reliably
- ◯ Unreliably

How much training will be required to collect the variable?

◯ Extensive
◯ Moderate
◯ Minimal

How much will this variable overlap with other variables?

◯ Significant overlap
◯ Minimal overlap
◯ Not applicable

Which variables will this variable overlap with?

____

How common is this variable abnormal?

◯ Frequently
◯ Less than half the time
◯ Rarely
◯ Not applicable

Other comments.

____

Burns

Should this variable be collected?

◯ Yes
◯ No

How strong is this variable as a predictor of severe illness in children?

◯ Strong
◯ Moderate
◯ Unlikely

Can this variable be reliably measured (i.e. inter- and intra-rater reliability)

◯ Highly reliable
◯ Moderately reliably
◯ Unreliably

How much training will be required to collect the variable?

◯ Extensive
◯ Moderate
◯ Minimal

How much will this variable overlap with other variables?

◯ Significant overlap
◯ Minimal overlap
◯ Not applicable

Which variables will this variable overlap with?

____

How common is this variable abnormal?

◯ Frequently
◯ Less than half the time
◯ Rarely
◯ Not applicable

Other comments.

____

Poisoning

Should this variable be collected?

- ◯ Yes
- ◯ No

How strong is this variable as a predictor of severe illness in children?

- ◯ Strong
- ◯ Moderate
- ◯ Unlikely

Can this variable be reliably measured (i.e. inter- and intra-rater reliability)

- ◯ Highly reliable
- ◯ Moderately reliably
- ◯ Unreliably

How much training will be required to collect the variable?

- ◯ Extensive
- ◯ Moderate
- ◯ Minimal

How much will this variable overlap with other variables?

- ◯ Significant overlap
- ◯ Minimal overlap
- ◯ Not applicable

Which variables will this variable overlap with? ________________________________

How common is this variable abnormal?

- ◯ Frequently
- ◯ Less than half the time
- ◯ Rarely
- ◯ Not applicable

Other comments. ____

General

Change in level of activity

Should this variable be collected?
- ◯ Yes
- ◯ No

How strong is this variable as a predictor of severe illness in children?
- ◯ Strong
- ◯ Moderate
- ◯ Unlikely

Can this variable be reliably measured (i.e. inter- and intra-rater reliability)
- ◯ Highly reliable
- ◯ Moderately reliably
- ◯ Unreliably

How much training will be required to collect the variable?
- ◯ Extensive
- ◯ Moderate
- ◯ Minimal

How much will this variable overlap with other variables?
- ◯ Significant overlap
- ◯ Minimal overlap
- ◯ Not applicable

Which variables will this variable overlap with? ______________________________

How common is this variable abnormal?
- ◯ Frequently
- ◯ Less than half the time
- ◯ Rarely
- ◯ Not applicable

Other comments.

____

Change in crying

Should this variable be collected?
- ◯ Yes
- ◯ No

How strong is this variable as a predictor of severe illness in children?
- ◯ Strong
- ◯ Moderate
- ◯ Unlikely

Can this variable be reliably measured (i.e. inter- and intra-rater reliability)
- ◯ Highly reliable
- ◯ Moderately reliably
- ◯ Unreliably

How much training will be required to collect the variable?
- ◯ Extensive
- ◯ Moderate
- ◯ Minimal

How much will this variable overlap with other variables?
- ◯ Significant overlap
- ◯ Minimal overlap
- ◯ Not applicable

Which variables will this variable overlap with? ______________________________

How common is this variable abnormal?

◯ Frequently
◯ Less than half the time
◯ Rarely
◯ Not applicable

Other comments.

____

Laboratory Results

Hemoglobin (g/dL)

Should this variable be collected?
- ◯ Yes
- ◯ No

How strong is this variable as a predictor of severe illness in children?
- ◯ Strong
- ◯ Moderate
- ◯ Unlikely

Can this variable be reliably measured (i.e. inter- and intra-rater reliability)
- ◯ Highly reliable
- ◯ Moderately reliably
- ◯ Unreliably

How much training will be required to collect the variable?
- ◯ Extensive
- ◯ Moderate
- ◯ Minimal

How much will this variable overlap with other variables?
- ◯ Significant overlap
- ◯ Minimal overlap
- ◯ Not applicable

Which variables will this variable overlap with? ________________________________

How common is this variable abnormal?
- ◯ Frequently
- ◯ Less than half the time
- ◯ Rarely
- ◯ Not applicable

Other comments.

____

Malaria blood smear

Should this variable be collected?
- ◯ Yes
- ◯ No

How strong is this variable as a predictor of severe illness in children?
- ◯ Strong
- ◯ Moderate
- ◯ Unlikely

Can this variable be reliably measured (i.e. inter- and intra-rater reliability)
- ◯ Highly reliable
- ◯ Moderately reliably
- ◯ Unreliably

How much training will be required to collect the variable?
- ◯ Extensive
- ◯ Moderate
- ◯ Minimal

How much will this variable overlap with other variables?
- ◯ Significant overlap
- ◯ Minimal overlap
- ◯ Not applicable

Which variables will this variable overlap with? ________________________________

How common is this variable abnormal?

◯ Frequently
◯ Less than half the time
◯ Rarely
◯ Not applicable

Other comments.

____

Blood sugar measurement

Should this variable be collected?

◯ Yes
◯ No

How strong is this variable as a predictor of severe illness in children?

◯ Strong
◯ Moderate
◯ Unlikely

Can this variable be reliably measured (i.e. inter- and intra-rater reliability)

◯ Highly reliable
◯ Moderately reliably
◯ Unreliably

How much training will be required to collect the variable?

◯ Extensive
◯ Moderate
◯ Minimal

How much will this variable overlap with other variables?

◯ Significant overlap
◯ Minimal overlap
◯ Not applicable

Which variables will this variable overlap with?

____

How common is this variable abnormal?

◯ Frequently
◯ Less than half the time
◯ Rarely
◯ Not applicable

Other comments.

____
