# Supplementary material for: Determining predictors of sepsis at triage among children under 5 years of age in resource-limited settings: A modified Delphi process
Source: PLoS One. 2019 Jan 28;14(1):e0211274. doi: 10.1371/journal.pone.0211274 (PMC6349330; doi:10.1371/journal.pone.0211274)
Supplement: S5 Appendix — (PDF) [file pone.0211274.s005.pdf]

Triage-Sepsis: Round 2 of the modified Delphi process

Dear colleagues,

Round 2 of Triage-Sepsis: modified Delphi survey

We would like to express our sincere thanks and appreciation for your participation in Round 1 of the survey to help determine potential predictor variables for sepsis in children between 2 months and 5 years of age in resource-limited settings. As a reminder, our study objective is to develop a prediction model to be used by front line workers during triage to identify sepsis in this age group.

We are pleased to present to you the following list of variables that have been accepted after the analysis of Round 1 results.

Patient Char.

Vitals

Neuro

Resp.

Cardio.

GI/Malnutrition

Age

Weight

A/V/P/U

Chest in-drawing

Capillary refill time

Dysentery

Gender

MUAC

Not being able to feed or drink anything

Apnea

Weak and fast pulse

Diarrhea

HIV status

Heart Rate

Convulsions (observed)

Increased respiratory effort

Cool peripheries

Vomiting

Duration of illness

Resp. Rate

Convulsions (reported)

Grunting

Pallor

Reduced urine production

Urgent referral status

SpO2

Irritability/restlessness

Difficulty breathing (observed)

Severe wasting

Time since last hosp.

Temperature

Not feeding well (reported)

Difficulty breathing (reported)

Peripheral edema

Fever

In Round 2, we would like to bring to your attention 4 new variables suggested by fellow colleagues to evaluate, and 12 variables previously presented in Round 1 for reconsideration. The group's aggregate responses for the 12 previous variables are reported for your reference. Please rate all variables according to the same three domains as from Round 1: predictive potential, measurement reliability, and the amount of training and resources required. The deadline of this survey is February 3rd, 2018.

We will analyze the results from this round to determine the final set of predictors to be included in the initial data collection. Concerns have been expressed that rare events would not be useful for prediction purposes. This issue will be addressed after the initial dataset is collected and analyzed and we have objective data on each predictor.

We would like to thank you once again for your participation in this survey. Your responses have been fundamental to selecting the predictors with the highest potential and practicality to identify sick children at triage in low-resource settings.

If there are any questions or concerns in completing this survey, please contact the principal investigator, Dr. Mark Ansermino at mansermino@cw.bc.ca.

Kind regards,

Pediatric Sepsis Data CoLab

https://bcchr.ca/pediatric-sepsis-data-colab

Part 1 - New Variables

New Variables

The following lists several new variables that have been suggested by experts to be included. Please rate them according to the three domains: predictive potential, measurement reliability, and level of training and/or resources required.

Jaundice

	High	Moderate	Minimal	Not Applicable
How strong is this variable as a predictor?	◯	◯	◯	◯
What is the likelihood that this variable can be reliably measured?	◯	◯	◯	◯
What would be the level of training or resources required to collect this variable?	◯	◯	◯	◯

Comments __________________________________

Number of previous hospital admissions

	High	Moderate	Minimal	Not Applicable
How strong is this variable as a predictor?	◯	◯	◯	◯
What is the likelihood that this variable can be reliably measured?	◯	◯	◯	◯
What would be the level of training or resources required to collect this variable?	◯	◯	◯	◯

Comments __________________________________

Surgical Predictors

Significant blood loss

	High	Moderate	Minimal	Not Applicable
How strong is this variable as a predictor?	◯	◯	◯	◯
What is the likelihood that this variable can be reliably measured?	◯	◯	◯	◯
What would be the level of training or resources required to collect this variable?	◯	◯	◯	◯

Comments __________________________________

Severe pain

	High	Moderate	Minimal	Not Applicable
How strong is this variable as a predictor?	◯	◯	◯	◯
What is the likelihood that this variable can be reliably measured?	◯	◯	◯	◯
What would be the level of training or resources required to collect this variable?	◯	◯	◯	◯

Comments ______________________________

Part 2 - Re-Evaluation of Round 1 Variables

Re-Evaluation of Round 1 Variables

Central cyanosis

20/26 experts selected "Yes" to collect this variable.
You answered "[round_1_arm_1][matrix_airway_breathing_4]".

The mode from the group response for each domain is as follows:
Predictability: High | Reliability: Moderate | Resources and/or training required: Moderate

You responded:
Predictability: [round_1_arm_1][airway_breathing_26] | Reliability: [round_1_arm_1][airway_breathing_27] | Resources and/or training required: [round_1_arm_1][airway_breathing_28]

Given the above results, please rate the variable again according to the three domains after your final consideration.

	High	Moderate	Minimal	Not Applicable
How strong is this variable as a predictor?	◯	◯	◯	◯
What is the likelihood that this variable can be reliably measured?	◯	◯	◯	◯
What would be the level of training or resources required to collect this variable?	◯	◯	◯	◯

Comments ______________________________

Nasal flaring

16/26 experts selected "Yes" to collect this variable.
You answered "[round_1_arm_1][matrix_airway_breathing_7]".

The mode from the group response for each domain is as follows:
Predictability: Moderate | Reliability: Moderate | Resources and/or training required: Moderate

You responded:
Predictability: [round_1_arm_1][airway_breathing_50] | Reliability:

Given the above results, please rate the variable again according to the three domains after your final consideration.

	High	Moderate	Minimal	Not Applicable
How strong is this variable as a predictor?	○	○	○	○
What is the likelihood that this variable can be reliably measured?	○	○	○	○
What would be the level of training or resources required to collect this variable?	○	○	○	○

Comments ______________________________

Stridor

19/26 experts selected "Yes" to collect this variable.
You answered "[round_1_arm_1][matrix_airway_breathing_11]".

The mode from the group response for each domain is as follows:
Predictability: Moderate | Reliability: Moderate | Resources and/or training required: Moderate

You responded:
Predictability: [round_1_arm_1][airway_breathing_82] | Reliability: [round_1_arm_1][airway_breathing_83] | Resources and/or training required: [round_1_arm_1][airway_breathing_84]

Given the above results, please rate the variable again according to the three domains after your final consideration.

	High	Moderate	Minimal	Not Applicable
How strong is this variable as a predictor?	○	○	○	○
What is the likelihood that this variable can be reliably measured?	○	○	○	○
What would be the level of training or resources required to collect this variable?	○	○	○	○

Comments ______________________________

Head bobbing/nodding

17/26 experts selected "Yes" to collect this variable.
You answered "[round_1_arm_1][matrix_airway_breathing_9]".

The mode from the group response for each domain is as follows:
Predictability: Moderate | Reliability: Moderate | Resources and/or training required: Moderate

You responded:
Predictability: [round_1_arm_1][airway_breathing_66] | Reliability: [round_1_arm_1][airway_breathing_67] | Resources and/or training required: [round_1_arm_1][airway_breathing_68]

Given the above results, please rate the variable again according to the three domains after your final consideration.

	High	Moderate	Minimal	Not Applicable
How strong is this variable as a predictor?	○	○	○	○
What is the likelihood that this variable can be reliably measured?	○	○	○	○
What would be the level of training or resources required to collect this variable?	○	○	○	○

Comments __________________________________

Reduced spontaneous movements/to stimulus

19/26 experts selected "Yes" to collect this variable.
You answered "[round_1_arm_1][matrix_neuro_13]".

The mode from the group response for each domain is as follows:
Predictability: Moderate | Reliability: Moderate | Resources and/or training required: Moderate

You responded:
Predictability: [round_1_arm_1][neuro_99] | Reliability: [round_1_arm_1][neuro_100] | Resources and/or training required: [round_1_arm_1][neuro_101]

Given the above results, please rate the variable again according to the three domains after your final consideration.

	High	Moderate	Minimal	Not Applicable
How strong is this variable as a predictor?	○	○	○	○
What is the likelihood that this variable can be reliably measured?	○	○	○	○
What would be the level of training or resources required to collect this variable?	○	○	○	○

Comments __________________________________

Neck pain/stiffness

19/26 experts selected "Yes" to collect this variable.
You answered "[round_1_arm_1][matrix_neuro_14]".

The mode from the group response for each domain is as follows:
Predictability: High | Reliability: Moderate | Resources and/or training required: Moderate

You responded:
Predictability: [round_1_arm_1][neuro_107] | Reliability: [round_1_arm_1][neuro_108] | Resources and/or training requried: [round_1_arm_1][neuro_109]

Given the above results, please rate the variable again according to the three domains after your final consideration.

	High	Moderate	Minimal	Not Applicable
How strong is this variable as a predictor?	○	○	○	○
What is the likelihood that this variable can be reliably measured?	○	○	○	○
What would be the level of training or resources required to collect this variable?	○	○	○	○

Comments ________________________

Bulging fontanelles

21/26 experts selected "Yes" to collect this variable.
You answered "[round_1_arm_1][matrix_neuro_15]".

The mode from the group response for each domain is as follows:
Predictability: High | Reliability: Moderate | Resources and/or training required: Moderate

You responded:
Predictability: [round_1_arm_1][neuro_115] | Reliability: [round_1_arm_1][neuro_116] | Resources and/or training required: [round_1_arm_1][neuro_117]

Given the above results, please rate the variable again according to the three domains after your final consideration.

	High	Moderate	Minimal	Not Applicable
How strong is this variable as a predictor?	○	○	○	○
What is the likelihood that this variable can be reliably measured?	○	○	○	○
What would be the level of training or resources required to collect this variable?	○	○	○	○

Comments ________________________

Sunken eyes

19/26 experts selected "Yes" to collect this variable.
You answered "[round_1_arm_1][matrix_dehydration_2]".

The mode from the group response for each domain is as follows:
Predictability: Moderate | Reliability: Moderate | Resources and/or training required: Moderate

You responded:
Predictability: [round_1_arm_1][dehydration_10] | Reliability: [round_1_arm_1][dehydration_11] | Resources and/or training required: [round_1_arm_1][dehydration_12]

Given the above results, please rate the variable again according to the three domains after your final consideration.

	High	Moderate	Minimal	Not Applicable
How strong is this variable as a predictor?	○	○	○	○
What is the likelihood that this variable can be reliably measured?	○	○	○	○
What would be the level of training or resources required to collect this variable?	○	○	○	○

Comments __________________________________

Reduced skin turgor

22/26 experts selected "Yes" to collect this variable.
You answered "[round_1_arm_1][matrix_dehydration_1]".

The mode from the group response for each domain is as follows:
Predictability: Moderate | Reliability: Moderate | Resources and/or training required: Moderate

You responded:
Predictability: [round_1_arm_1][dehydration_2] | Reliability: [round_1_arm_1][dehydration_3] | Resources and/or training required: [round_1_arm_1][dehydration_4]

Given the above results, please rate the variable again according to the three domains after your final consideration.

	High	Moderate	Minimal	Not Applicable
How strong is this variable as a predictor?	○	○	○	○
What is the likelihood that this variable can be reliably measured?	○	○	○	○
What would be the level of training or resources required to collect this variable?	○	○	○	○

Comments __________________________________

Oral thrush

20/26 experts selected "Yes" to collect this variable.
You answered "[round_1_arm_1][matrix_malnutr_4]".

The mode from the group response for each domain is as follows:
Predictability: High | Reliability: Moderate | Resources and/or training required: Moderate

You responded:
Predictability: [round_1_arm_1][malnutr_26] | Reliability: [round_1_arm_1][malnutr_27] | Resources and/or training required: [round_1_arm_1][malnutr_28]

Given the above results, please rate the variable again according to the three domains after your final consideration.

	High	Moderate	Minimal	Not Applicable
How strong is this variable as a predictor?	○	○	○	○
What is the likelihood that this variable can be reliably measured?	○	○	○	○
What would be the level of training or resources required to collect this variable?	○	○	○	○

Comments ______________________________

Cough

18/26 experts selected "Yes" to collect this variable.
You answered "[round_1_arm_1][matrix_infection_2]".

The mode from the group response for each domain is as follows:
Predictability: Minimal | Reliability: Moderate | Resources and/or training required: Minimal

You responded:
Predictability: [round_1_arm_1][infection_10] | Reliability: [round_1_arm_1][infection_11] | Resources and/or training required: [round_1_arm_1][infection_12]

Given the above results, please rate the variable again according to the three domains after your final consideration.

	High	Moderate	Minimal	Not Applicable
How strong is this variable as a predictor?	○	○	○	○
What is the likelihood that this variable can be reliably measured?	○	○	○	○
What would be the level of training or resources required to collect this variable?	○	○	○	○

Comments ______________________________

Systolic blood pressure

20/26 experts selected "Yes" to collect this variable.
You answered "[round_1_arm_1][matrix_vitals_5]".

The mode from the group response for each domain is as follows:
Predictability: High | Reliability: Moderate | Resources and/or training required: Moderate

You responded:
Predictability: "[round_1_arm_1][vitals_43]" || Reliability: "[round_1_arm_1][vitals_44]" || Resources and or training required: "[round_1_arm_1][vitals_45]"

Given the above results, please rate the variable again according to the three domains after your final consideration.

	High	Moderate	Minimal	Not Applicable
How strong is this variable as a predictor?	◯	◯	◯	◯
What is the likelihood that this variable can be reliably measured?	◯	◯	◯	◯
What would be the level of training or resources required to collect this variable?	◯	◯	◯	◯

Comments __________________________________
